# Input-output efficiency, productivity dynamics, and determinants in western China’s higher education: A three-stage DEA, global Malmquist index, and Tobit model approach

**DOI:** 10.1371/journal.pone.0325901

**Published:** 2025-06-11

**Authors:** Rui Guo, Meng Ye

**Affiliations:** 1 School of Foreign Languages, Dalian Maritime University, Dalian, Liaoning, China; 2 College of Public Administration and Humanities, Dalian Maritime University, Dalian, Liaoning, China; Universidade de Lisboa Instituto Superior Tecnico, PORTUGAL

## Abstract

Amid rapid globalization and the expansion of the knowledge economy, enhancing input-output efficiency of higher education is essential for regional development and national competitiveness. In resource-constrained regions like western China, it is of utmost significance to obtain efficiency gains by accurately assessing this efficiency and identifying its determinants, which are crucial for balanced regional development and educational equity. This study employs a comprehensive research methodology, combining a three-stage Data Envelopment Analysis, the global Malmquist productivity index, and a Tobit model to systematically evaluate input-output efficiency, productivity dynamics, and factors influencing technical efficiency in higher education across 12 provinces in western China from 2010 to 2022. The findings reveal that environmental factors and random errors significantly affect the input-output efficiency of higher education in western China, resulting in an overestimation of overall efficiency. Adjusted values for technical efficiency, pure technical efficiency, and scale efficiency are 0.8510, 0.9761, and 0.8707, respectively. Although most western provinces demonstrate relatively high pure technical efficiency, deficiencies in scale efficiency reduce overall technical efficiency, particularly in Tibet, Qinghai, and Ningxia. Total factor productivity exhibits a modest annual growth rate of 1.05%, driven by both technical efficiency advancements and technological progress, with technical efficiency change—primarily via enhanced scale efficiency—acting as the main contributor. Moreover, human capital structures and educational funding allocations significantly impact technical efficiency. Specifically, the proportion of full-time faculty with senior or associate senior academic titles and per-student education expenditure have a significantly negative influence on technical efficiency, whereas the share of operating expenses in total higher education expenditure and the proportion of employees with at least a college education are positively correlated with technical efficiency. This study offers empirical evidence to inform the formulation of targeted policies for the development of higher education in western China.

## 1. Introduction

Higher education serves as a vital indicator of a nation’s developmental level and future potential, as both technological advancement and economic growth are fundamentally contingent upon the caliber of its higher education. As globalization deepens and the knowledge economy accelerates, higher education has emerged as a critical pillar in enhancing national competitiveness. It fosters economic growth, primarily by cultivating skilled talent and generating human capital [1]. It functions as a core hub for scientific research and technological innovation, which bolsters national competitiveness through knowledge accumulation and technology transfer [2–4]. It promotes social equity, creates opportunities for upward mobility, and contributes to cultural transmission and innovation. In addition, its social service function supports local development and addresses societal needs via industry-academia-research collaboration. In essence, higher education is a primary engine driving holistic economic, social, and cultural development.

The realization of these multidimensional goals in higher education depends on enhancing input-output efficiency, ensuring that limited resources yield maximum socioeconomic benefits. Efficient resource allocation can minimize costs, optimize funding distribution, and redirect savings to areas such as research, scholarships, and faculty development—ultimately improving educational quality, learning environments, and faculty capacity, while also enhancing institutional effectiveness and broader societal advancements [5–7]. Moreover, optimizing resource efficiency also enables higher education institutions (HEIs) to accommodate more students and expand access to education, particularly vital in developing countries where educational resources are scarce yet demand remains high. Through effective resource management, educational institutions can uphold standards while accommodating more students, reducing inequities in educational access [8]. Enhancing resource allocation efficiency in HEIs is thus critical to sustainable development and central to management reforms worldwide, where performance accountability is increasingly emphasized [9–10]. Consequently, an increasing number of countries are placing greater emphasis on optimizing resource efficiency within HEIs [11], thereby necessitating the evaluation of resource utilization effectiveness [12–13].

Being the second-largest economy globally, China is recognized as a significant growth pole in the global higher education landscape. Recent years have witnessed substantial investments by the Chinese government in higher education, aimed at elevating the international standing of Chinese universities [14]. China’s higher education has now progressed to a phase of universalization, with a continuously expanding scale. By 2022, China’s population with higher education had reached 240 million. By 2023, China hosted a total of 3,074 HEIs, including 1,242 regular undergraduate institutions, 33 undergraduate vocational colleges, 1,547 higher vocational (junior college) institutions, and 252 adult education institutions. Additionally, 233 research institutions were authorized to confer graduate degrees. Total enrollment in higher education across all forms reached 47.63 million, corresponding to a gross enrollment rate of 60.2%. Nonetheless, challenges persist amid this expansion. Demographic shifts, intensifying competition for student enrollment, and adjustments in national funding policies compel HEIs to confront the pressure of optimizing resource allocation, improving input-output efficiency, and enhancing educational quality [15].

Western China encompasses 12 provincial-level administrative divisions, covering approximately 71% of China’s total land area. Despite its vast geographical expanse, the region remains economically underdeveloped and educationally underserved compared to other parts of the country, while also serving as a primary area for multi-ethnic residence and cultural convergence. Higher education in western China plays an indispensable role within the broader framework of China’s higher education system. First, its status and advancement profoundly impact regional revitalization and coordinated development. Second, promoting higher education in this region fosters educational equity, helping to narrow the urban-rural and regional educational disparities. Finally, it is indispensable for promoting national unity and preserving diverse cultural heritage. Given the unique mission of higher education in the west and the dual constraints of limited educational input and output, the Chinese government has issued more than twenty policy documents following the launch of the Western Development Strategy in 1999 to promote the growth of higher education in this region. Among these, the *Opinions on Further Promoting Partner Assistance to Higher Education Institutions in the Western Region*, the *Central and Western Higher Education Revitalization Plan (2012–2020)*, and the *Opinions on the Revitalization of Higher Education in the Central and Western Regions in the New Era* stand out as pivotal policy frameworks. Based on these policy milestones, higher education in western China unfolds in three stages: 2001–2012, marked by scale expansion and external support; 2013–2019, focused on quality improvement through a blend of external assistance and internal capacity building; and from 2020 onward, an era emphasizing endogenous and high-quality development. Policy support has enabled significant progress in western higher education, evident in expanded enrollment, increased coverage, improved basic infrastructure, and enhanced teaching and research capacity. The number of HEIs in western China increased from 298 in 2001–850 in 2022, accounting for 27.87% of all institutions nationwide. Nonetheless, western higher education still lags behind its eastern counterparts in terms of quality and structure, adaptation to socioeconomic needs, and contributions to economic growth [16]. Moreover, constrained by unfavorable geographic factors, talent attrition, and suboptimal resource utilization efficiency, the competitiveness of western higher education remains inadequate.

The motivation for this study is based on both academic and practical considerations. From an academic standpoint, existing research on higher education efficiency has two main limitations: First, it overlooks the regional disparities and multi-ethnic context of western China. Most studies focus on China as a whole or on the economically developed eastern regions, while research on the efficiency of higher education in the economically underdeveloped and multi-ethnic western regions is relatively scarce. In particular, the impact of regional differences, economic imbalances, and multi-ethnic cultural backgrounds on efficiency evaluations has not been adequately addressed. Second, the methods used for efficiency evaluation are often overly simplistic, and cross-period comparisons face challenges. The majority of studies rely solely on methods such as SFA or DEA, lacking a synthesis of different approaches. Furthermore, the reference technologies used in these studies often employ production possibility sets from different time periods, which limits the accuracy of efficiency assessments and the comparability of results across periods. From a practical perspective, the quality of higher education in western China is crucial to the nation’s overall competitiveness, regional economic development, social stability and educational equity. Over the past decade and more, China’s higher education has shifted from scale expansion to a focus on intrinsic, high-quality development, emphasizing educational quality, resource utilization efficiency, and structure optimization. To elevate Chinese higher education and promote balanced, high-quality growth, it is of paramount importance to address the shortcomings of higher education in western China, such as unequal resource allocation, low development quality, and insufficient competitiveness. Given the overall scarcity of educational resources, enhancing the input-output efficiency of higher education in western China has become critical for overcoming developmental bottlenecks, which necessitates an accurate estimation of the efficiency in this area. In response, this study has three primary research objectives: (1) to assess the overall and provincial input-output efficiency of higher education in western China; (2) to analyze productivity changes from the perspective of total factor productivity (TFP) in both regional and provincial contexts; and (3) to identify the key factors influencing input-output efficiency of higher education in western China. This study aims to provide an innovative perspective on higher education efficiency research, reconcile the scarcity of higher education resources in western China with existing demands, and promote equitable access to higher education. Furthermore, it seeks to offer empirical support for the government in formulating more precise and scientifically grounded higher education policies, enhancing the effectiveness of policy implementation, and advancing the continuous deepening of educational reforms.

This study first employs a three-stage Data Envelopment Analysis (DEA) model proposed by Fried et al. [17], utilizing provincial panel data from 2010 to 2022 to gauge and decompose the input-output efficiency of higher education across the 12 provinces (including municipalities and autonomous regions) in western China. Second, it employs the global Malmquist productivity index to decompose TFP and examine the dynamic trends in productivity and their driving forces within western higher education. Finally, the study utilizes a Tobit model to determine the factors and mechanisms influencing the efficiency of higher education in western China. This study lays a scientific foundation for optimizing resource allocation and advancing input-output efficiency of higher education across western China. Furthermore, it aspires to serve as a valuable reference for other underdeveloped regions’ higher education, thereby contributing broadly to reducing regional disparities and promoting balanced educational advancement.

The study makes three methodological contributions and three empirical contributions to the current literature. First, the study integrates multiple research methods to achieve a multidimensional evaluation of higher education efficiency, overcoming the limitations of traditional, singular evaluation approaches. The three-stage DEA method combines the advantages of both parametric and non-parametric models, accurately estimating the net efficiency of higher education by eliminating the influence of external environmental factors and random noise. This method addresses potential biases in efficiency calculations inherent in traditional DEA approaches, enhancing the reliability and scientific rigor of efficiency assessments. Second, this study innovatively combines global benchmark technology (global DEA) with the global Malmquist index to construct a unified cross-period reference set. The conventional Malmquist index constructs production technology sets using single-period cross-sectional input and output data from decision-making units (DMUs), which can result in productivity changes that lack robustness and continuity [18–19]. This methodological advancement resolves the critical issue in previous research, where efficiency and productivity were not comparable across periods due to changes in standards of reference technology. The unified reference technology provides a solid theoretical foundation and methodological tool for the cross-period analysis and dynamic evaluation of higher education efficiency. Third, taking into account the pronounced regional disparities in western China, this study introduces a systematic three-dimensional theoretical framework. It identifies socioeconomic conditions, human capital levels, and educational funding structures as core influencing factors and comprehensively examines the complex interactions of both external environments and internal resource allocation on input-output efficiency of higher education. This framework broadens the scope of efficiency research and offers a new theoretical perspective for evaluating higher education efficiency in resource-constrained, economically underdeveloped, and ethnically diverse regions. It also addresses a gap in previous studies, which have insufficiently focused on regional differences and the factors affecting efficiency. Fourth, the study is the first to demonstrate that the external environment significantly affects both the input-output technical efficiency and scale efficiency of higher education in regions such as Tibet and Qinghai in western China. Failure to account for these external influences can lead to substantial overestimation of efficiency. This finding fills a gap in the existing literature regarding the analysis of external environmental factors and underscores the necessity of considering such interference effects of external factors when conducting efficiency evaluations in regions with significant economic and social heterogeneity. Fifth, the study reveals the heterogeneous impact mechanisms of both internal and external human capital on the input-output efficiency of higher education in western China. External human capital levels (such as the proportion of employees with college education or above) have a significant positive effect, whereas internal human capital levels (such as the proportion of full-time faculty with senior academic titles) have a suppressive effect. Notably, the study finds that simply increasing the proportion of professors and associate professors in HEIs has a limited impact on improving the input-output efficiency of higher education. This conclusion challenges the conventional belief that higher academic titles necessarily enhance efficiency, suggesting that attention should be paid to the synergy between the quality and distribution structure of human capital in the allocation of higher education resources. This finding enriches the theoretical research on the optimal allocation of higher education resources and provides new decision-making insights for improving educational efficiency in resource-constrained areas. Finally, this study identifies the primary pattern for improving the input-output efficiency of higher education in western China: enhancing scale efficiency leads to improvements in technical efficiency, thereby driving growth in total factor productivity. This finding provides a practical and actionable pathway for enhancing input-output efficiency of higher education in western China, as well as in other developing countries or regions with resource constraints, offering valuable policy guidance.

The remainder of the study is structured as follows: Section 2 offers an extensive review of the literature. Section 3 describes the research methodologies in detail. Section 4 introduces the model variables, data sources, as well as descriptive statistics. Section 5 presents the empirical results and an in-depth discussion. Finally, Section 6 concludes the study and outlines the policy implications.

## 2. Literature review

### 2.1 Evaluation methods for higher education efficiency

Higher education efficiency has consistently been a primary concern in educational economics, with an emphasis on evaluating the relationship between scarce resource inputs and corresponding outputs. Methodologically, efficiency assessment approaches are broadly categorized into parametric and non-parametric techniques [20], with Stochastic Frontier Analysis (SFA) being the most widely applied among parametric methods [21–26], and DEA dominating the non-parametric sphere [27–28].

SFA involves constructing a cost or production function and estimating frontier parameters before application. However, determining a suitable functional form remains challenging, as assumptions about the selected functional form can substantially impact estimation results, leading to variability and potential inconsistencies in outcomes. At present, no definitive method or standard exists for identifying the “correct” or “appropriate” functional form [29]. Additionally, SFA is generally constrained to single-output scenarios, complicating its application in contexts with multiple inputs and outputs. This limitation is pertinent in higher education, where universities typically operate with complex, multi-dimensional input-output relationships, making the selection of an appropriate production function a non-trivial and often ambiguous task [30]. Therefore, while SFA serves as a valuable methodological tool, its applicability in multi-output systems, coupled with the complexities of selecting an appropriate functional form, continues to pose significant challenges.

In contrast, DEA circumvents the need for stringent assumptions regarding the functional form between inputs and outputs, while also avoids the subjective weighting of these variables [31–34]. Moreover, DEA’s dimensional invariance renders it particularly suitable for analyzing systems characterized by multiple inputs and outputs [35–40]. Based on the aforementioned features, DEA has been widely employed as a benchmarking tool across various sectors, including banking, healthcare, agriculture, transportation, and education [41]. Building on the groundbreaking work of Charnes et al. [27], DEA’s utility in evaluating educational systems, especially in higher education, has been extensively validated [42–55]. DEA method offers two key advantages in evaluating efficiency of HEIs. It allows for a multidimensional assessment of university performance, revealing critical insights into various aspects of efficiency [13]. Besides, DEA’s flexibility, derived from its non-reliance on a predefined production function, enables the pinpointing of areas for enhancement and the exploration of developmental potential.

DEA has developed numerous extended models, with the three-stage DEA model gaining recognition in higher education and other academic domains in recent years. This model’s enhanced capability to account for environmental variables and random noise generates efficiency estimates that reflect real-world conditions more accurately. Fuentes et al. [56] employed the three-stage DEA model to assess the technical efficiency of the learning-teaching process in higher education, finding contextual variables exerting a significant influence. Wu et al. [29] adopted the same model to measure the performance of HEIs across 31 provinces in China, effectively addressing measurement errors and environmental variables. Xue et al. [57] combined the three-stage DEA and the Malmquist index to measure the efficiency of scientific research in Chinese universities under the Ministry of Education, while incorporating external environmental factors. Their findings revealed that the three-stage DEA model provided more precise efficiency measurements for scientific research inputs and outputs than traditional DEA methods, with scale optimization identified as the main internal driver of efficiency enhancement. Beyond the education sector, the three-stage DEA has found widespread use in efficiency evaluations across fields, including energy efficiency [58], industrial eco-efficiency [59], green productivity growth in manufacturing [60], and cultural industry investment efficiency [61]. Across these studies, environmental variables consistently demonstrated significant influences on efficiency outcomes, underscoring the three-stage DEA model’s value for robust and accurate efficiency assessments.

### 2.2 Analytical units for higher education efficiency

Current research on higher education efficiency using the DEA method predominantly focuses on two main analytical units. The first, most prevalent approach adopts a micro-level perspective, treating universities or institutions as DMUs to examine the efficiency of teaching, research, and other institutional functions. This approach compares performance either across different universities or among various departments within individual institutions. The second approach takes a macro-level perspective, considering countries or regions as DMUs. This research compares HEI efficiency across different nations or regions within a single country, offering broader insights into national or regional educational systems.

Research considering universities as DMUs: Athanassopoulos and Shale [62] conceptualized cost and outcome efficiency to provide deeper understanding of university operations. The UK has been a pioneer in research on higher education efficiency, amassing numerous studies. Flegg et al. [63] examined the technical efficiency of 45 UK universities from 1980/81–1992/93, revealing that improvements in technical efficiency were largely driven by gains in pure technical efficiency, while scale efficiency had a smaller impact. Johnes [64] analyzed the efficiency of over 100 HEIs in the UK, finding that the average levels of both technical and scale efficiency were considerable, though marked disparities existed between the most and least efficient institutions. Casu and Thanassoulis [65] focused on cost efficiency within UK universities’ central administration, highlighting challenges in defining assessment units and input-output relationships. Glass et al. [66] calculated efficiency scores to inform policy evaluation and potential funding allocation within the higher education system in the UK. Bradley et al. [67] evaluated the efficiency and productivity changes across approximately 200 further education institution in the UK from 1999 to 2003. Beyond the UK, research on higher education efficiency extends to European countries, Australia, the United States, Latin America, and several Asian regions. In Germany, Kempkes and Pohl [30] utilized DEA and SFA to examine the efficiency of 72 public universities, concluding that eastern German universities outperformed their western counterparts in TFP change, despite lower average efficiency scores. Başkaya and Klumpp [68] conducted a comparative analysis of the input-output efficiency between public and private universities in Germany. In Greece, Katharaki and Katharakis [69] evaluated the efficiency of 20 public universities using performance indicators, DEA, and econometric techniques, identifying inefficiency in human resource management. In Poland, Nazarko and Šaparauskas [13] used a CCR-CRS output-oriented DEA model to analyze potential inputs, outputs, and environmental factors in 19 universities of technology, finding that institutional outcomes contributed more to enhancing output efficiency than the volume of resources available. In Italy, Guccio et al. [70] applied bootstrapped DEA algorithms to evaluate the effects of university reform on educational efficiency, showing progressive efficiency gains. In Australia, some scholars [71–72] utilized DEA to measure the relative, technical, and scale efficiency of universities, reporting relatively high overall efficiency levels. In the United States, Hirao [73] measured efficiency of the top 50 public and private business schools in 2006, noting that while both types demonstrated high technical efficiency, public institutions lagged in scale and overall efficiency. In Colombia, Visbal-Cadavid et al. [74] applied CCR, BCC, and SBM output-oriented DEA models to examine technical, pure technical, scale, and mix efficiency across public universities, further examining productivity changes from 2011 to 2012 using the Malmquist index. Navas et al. [75] investigated the efficiency of Colombian HEIs using a cross-efficiency DEA method, finding that certain institutions demonstrated high efficiency in teaching or employment, whereas others excelled in research. In Thailand, Kantabutra and Tang [76] employed DEA models focused on teaching and research efficiency to assess faculty-level efficiency at public universities, comparing performance differences between government-run and autonomous universities.

Some studies target the efficiency evaluation of various faculties and academic departments within universities. For instance, Leitner et al. [77] assessed the efficiency of natural sciences and engineering departments at Austrian HEIs with two input variables and 12 output variables, finding that both scale and pure technical effects contributed to the economic efficiency. Gimenez and Martinez [78] proposed a cost efficiency analysis model within the DEA framework and applied it to assess the efficiency of various departments at the Autonomous University of Barcelona.

Research considering countries or regions as DMUs: Studies of this type are relatively scarce, with the majority focusing on cross-national comparative analyses. Agasisti and Pohl [79] performed a comparative evaluation of the efficiency of public universities in Italy and Germany during the 2001–2007 period, finding that while German universities tended to be more efficient overall, Italian institutions exhibited significant efficiency improvements during this period. Wolszczak-Derlacz [80] evaluated the efficiency of HEIs in Europe and the United States, accounting for national-level heterogeneity. Agasisti et al. [81] conducted a meta-frontier analysis to compare the productivity between elite universities in China and Europe, revealing that Chinese universities demonstrated more rapid productivity growth than their European peers. Dincă et al. [82] assessed the educational efficiency of 28 European Union member states, concluding that the older member states consistently outperformed newer ones. Chen et al. [83] conducted a comprehensive assessment of educational efficiency and its dynamic changes across 29 major countries using the super-SBM model and the Malmquist index, highlighting substantial disparities in average educational efficiency, with Japan achieving the highest ranking and Norway the lowest. Moreover, the study identified technological progress as the key contributor to the growth of TFP in education. Jean-Pascal and Nicolas [84] measured the efficiency of educational diffusion and research productivity in the United States, considering geographical disparities between urban and rural regions, alongside distinctions between public and private universities. Their results indicated that urban universities placed greater emphasis on educational quality, while public universities demonstrated higher educational efficiency but lower research efficiency. Liao et al. [85] assessed the efficiency of educational resource use, productivity dynamics, and regional technological disparities across 35 European countries and four regions, using DEA Super-SBM, meta-frontier analysis, and the Malmquist productivity index.

In studies on China, several analyses have evaluated the efficiency of provincial-level HEIs and university research performance. Zhang et al. [86] performed a provincial-level analysis of dynamic changes in Chinese HEIs, proposing a DEA-based framework for optimizing the distribution of enrollment quotas. Sun et al. [16] undertook a dynamic evaluation of higher education efficiency across 30 provinces of mainland China, employing a meta-frontier Super-SBM model to explore regional disparities. The findings indicated that efficiency change was the primary driver of performance, with significant technology gap variations among the eastern, central, and western regions. In light of these disparities, the study recommended that the Chinese government should prioritize reducing regional imbalances in higher education. Liu et al. [87] applied the DEA super-SBM model, the Malmquist productivity index, and meta-frontier analysis to evaluate the efficiency, productivity changes, and technology gaps within the provincial higher education systems of China. Their findings indicated that provinces with lower literacy levels had higher efficiency than middle and high-literacy provinces, and efficiency change was the primary driver of productivity growth in higher education rather than technological advancements. Chen et al. [88] adopted a super-efficiency DEA model alongside the Malmquist index to assess the resource allocation efficiency of Chinese agricultural universities, also capturing dynamic evolution and identifying efficiency drivers.

Research on China’s higher education places a pronounced emphasis on research efficiency. For instance, Johnes and Yu [89] investigated the relative efficiency of research output across 109 regular universities in 2003 and 2004. Their study indicated that average research efficiency was greater in comprehensive universities than in specialized institutions, and in universities situated in coastal regions as opposed to those located in western China. Xiong et al. [90] and Ng & Li [91] measured the research performance of Chinese HEIs in humanities and social sciences, further proposing an efficiency decomposition by Malmquist productivity index. Wang et al. [92] adopted DEA-BCC model to evaluate and contrast scientific input-output efficiency of research-oriented universities in the Chengdu-Chongqing urban agglomeration, finding a marginal increase in average research efficiency. However, the study noted that the influence of scale on overall efficiency remained minimal, and there was a prominent misalignment between research themes, funding allocation, and human resources in the sampled universities. Wang and Hu [93] examined the overall and stage-specific research efficiencies of 18 HEIs in Jiangsu province.

### 2.3 Factors influencing higher education efficiency

With respect to methodologies for identifying the determinants of educational efficiency, scholars have used Ordinary Least Squares (OLS) [94], bootstrapped truncated regression [95–97], and the Tobit model [30,98]. Of these, the Tobit model is the most commonly adopted and considered highly robust, particularly advantageous for handling censored data and providing straightforward estimation results.

The input-out efficiency of HEIs is influenced by external and internal factors, broadly divided into external environmental influences and internal structural determinants [99–101]. Key external factors include regional economic development [30] and financial resources [102], while the primary internal factor is human capital [103–104]. Together, these factors critically affect HEIs’ operational efficiency and provide insights into institutional performance variations.

Regional economic development: Wolszczak-Derlacz [80] highlighted regional heterogeneity in the allocation of higher education resources across the United States and Europe, with affluent regions showing notably higher efficiency in resource use. Berbegal-Mirabent et al. [105] measured the resource allocation efficiency of Spanish universities and highlighted regional economic activity as a key determinant. Similarly, Kempkes and Pohl [30] found a positive correlation between per capita GDP and higher education efficiency in German HEIs. Chen et al. [106] further demonstrated a significant positive relationship between regional economic environment and both TFP and technical efficiency in China’s “double first-class” universities. Consistent with these findings, some studies argued that regional economic development provided strong financial support for enhancing university efficiency [107–108]. Nevertheless, other studies suggest that neither per capita GDP nor regional economic strength exerts a significant or clearly synchronous impact on higher education efficiency [101,109]. Moreover, when resource allocation structure is irrational, further regional economic development may exacerbate issues such as redundant inputs or inadequate outputs [110–111].

Financial resources: Grounded in public goods theory, universities are recognized as key nonprofit institutions, with government financial support serving as a critical material foundation to alleviate resource constraints and foster institutional development [112–113]. Robst [114], utilizing the SFA model in his analysis of higher education cost-efficiency, identified a significant positive relationship between government appropriations and cost efficiency. Similarly, Klumpp [115], through the application of the DEA–Malmquist model, confirmed that increased funding inputs positively influenced the international university ranking. Wolszczak-Derlacz and Parteka [101] demonstrated that a higher proportion of external funding significantly enhanced institutional efficiency in their study of 259 public HEIs across seven European countries. Conversely, Chen et al. [106] reported an inverse relationship between regional financial support and university TFP. Fadda et al. [116] further argued that government funding often followed a differentiated approach, which might result in opportunistic behavior or the Matthew effect, undermining HEIs’ incentives to optimize resource allocation efficiency.

Human capital: The faculty title structure is a widely recognized indicator for assessing the operational and developmental status of HEIs [117]. Chen and Yang [118] argued that teachers with senior academic titles significantly contributed to talent cultivation and scientific research due to their advanced expertise and extensive research resources, thereby enhancing university efficiency. In the same vein, Salas-Velasco [97], through a two-stage semi-parametric approach with single and double bootstrap procedures, found that universities with a higher proportion of professors among their academic staff exhibited greater efficiency. Chen and Shu [55], using the super-efficiency DEA model, Malmquist index, and Tobit-DEA model, analyzed the determinants of scientific and technological innovation efficiency. Their findings suggested that a higher proportion of senior full-time faculty, an optimized resource allocation structure, as well as government support positively influenced innovation efficiency. In the Chinese academic community, numerous studies have confirmed that the proportion of full-time faculty with senior ranks significantly enhanced the technical efficiency of HEIs [103,119]. However, studies focusing on China’s western regions indicate that the proportion of employees with tertiary education or above, as a proxy for human capital, negatively affects the effectiveness of public investment and the higher education efficiency in these regions [120–121].

Other factors: Oliviera and Santos [95] analyzed the efficiency of 42 public schools in Portugal and found that regional unemployment rate exerted a negative influence on school efficiency. Wolszczak-Derlacz and Parteka [101] concluded that a higher proportion of female faculty members significantly enhanced institutional efficiency. Hendin [122] suggested that policy implementation was essential in facilitating university resource acquisition. The government utilizes policy instruments to bolster higher education by redistributing resources to universities in less developed regions and particular kinds of institutions [123]. However, Nisar [124] argued that universities’ responses to policies often followed a nonlinear and unpredictable pattern, making the concrete impact of policy enactment difficult to predict. Additionally, several Chinese studies demonstrate that indicators such as the proportion of operating expenses to total educational expenditures, regional industrial development levels, and per-student education funding exhibit significant positive or negative correlations with the overall efficiency of higher education [119,125,126].

Drawing on prior research, this study offers supplementary contributions, improves methodologies, and provides novel insights from a research perspective. (1) Expanding the research perspective. The majority of previous studies concentrates on macro- or micro-level analyses of higher education efficiency. Macro-level research typically compares efficiency disparities across different countries or regions, while micro-level studies concentrate on efficiency differences among universities or departments. However, there is a notable scarcity of research at the meso-level, particularly regarding the higher education efficiency across the four major regions of China (Eastern, Central, Western, and Northeastern). Consequently, this study fills the gap in meso-level research by undertaking a thorough analysis of the input-output efficiency of higher education in western China, further uncovering internal disparities and their underlying causes. This analysis aims to provide policy recommendations to mitigate the deficiencies in China’s higher education system and promote more balanced development. (2) Enhancing the research methodology. Most existing studies employ traditional DEA models to evaluate higher education efficiency. In contrast, the three-stage DEA model offers significant advantages over traditional models by effectively eliminating the influence of environmental factors and random noise on efficiency, thereby yielding more accurate efficiency estimates. However, the application of the three-stage DEA method in the context of regional higher education research in China has been insufficient. Therefore, this study utilizes the three-stage DEA model to improve the precision of efficiency estimates for higher education in western China, ultimately providing more accurate, effective, and targeted recommendations for regional higher education management. (3) Supplementing dynamic efficiency research. Current literature predominantly focuses on static efficiency in higher education, with limited attention to dynamic changes in efficiency. As Parteka and Wolszczak-Derlacz [15] pointed out, dynamic changes in HEIs’ productivity have only been marginally analyzed. Because the DEA-CCR, DEA-BCC, and network DEA models are predominantly designed for assessing static efficiency, they are ill-suited for capturing the dynamics of efficiency changes over time [127–128]. This insurmountable shortcoming renders singular models, such as SFA or DEA, insufficient to fully address the increasingly complicated and precise computational demands in contemporary higher education [106]. As a result, scholars have begun to augment these models by incorporating Malmquist index models [129–130]. This study advances the literature by incorporating the global Malmquist index to systematically analyze efficiency changes over time, which provides a thorough grasp of the dynamic trajectories and underlying drivers of higher education efficiency in western China. (4) Providing new evidence on discrepancies in efficiency influencing factors. There is considerable divergence in the academic discourse surrounding the factors influencing higher education efficiency. Existing studies primarily focus on factors such as regional economic development levels, government financial support, and the internal human capital structure of universities, often in the context of developed countries or regions. However, for the relatively underdeveloped and resource-constrained western China, it is crucial to investigate whether the influencing factors and mechanisms discussed in existing literature are consistent with those in developed regions. This study conducts a multi-faceted and comprehensive analysis of the factors affecting higher education efficiency, taking into account the unique characteristics of western China, thereby providing new evidence regarding the determinants of higher education efficiency in western China.

## 3. Methodology

### 3.1 Three-stage DEA model

Data Envelopment Analysis (DEA) is proposed by American operations researchers Charnes et al. in 1978, aimed at quantifying the relative efficiency of DMUs with multiple inputs and outputs of the same type [27]. This non-parametric approach uses linear programming techniques to systematically assess the relative efficiency of DMUs [131]. The DEA model spatially maps input-output points, establishing boundaries based on minimal input or maximal output, known as the best practice production frontier. This framework functions as a benchmark for evaluating the distance of other points from the frontier, thereby embodying the concept of relative efficiency by measuring the proximity of input-output points to the best practice production frontier [97].

However, the traditional DEA model has limitations in efficiency evaluation, such as its inability to account for external environmental factors, random noise, and its reliance on static analysis [29]. Fried et al. [17] explain that the efficiency of DMUs can be affected by three distinct phenomena: the efficiency with which production activities are organized by management, the characteristics of the environment in which production activities are carried out, and the stochastic impacts of luck, omitted variables, and related phenomena, which can be captured in a random error term in a regression-based evaluation of DMUs’ efficiency. To accurately disentangle the three influences—managerial inefficiencies, environmental effects, and statistical noise—comprehensive data on environmental characteristics, inputs, and outputs are essential. Moreover, capturing the stochastic nature of luck requires the use of stochastic modeling approaches; however, it is noteworthy that most DEA models, particularly those adopted in previous studies, are deterministic, thus failing to adequately address the inherent stochastic complexities of DMU efficiency assessments. In light of this, Fried et al. [17] put forward a three-stage DEA model in 2002, building upon the traditional DEA framework.

Fried’s approach consists of three stages: In the first stage, the traditional DEA model is applied to calculate the initial efficiency scores of DMUs along with various input slacks. The second stage establishes a SFA regression model [21–22], where input slacks are the dependent variables and external environmental factors are the independent variables. This model adjusts the inputs from the first stage to account for environmental influences and random errors, ensuring that the adjusted DMUs are subjected to identical external environment and stochastic components. In the third stage, the adjusted inputs from the second stage, along with the original outputs, are utilized to recompute the efficiency scores of each DMU using the traditional DEA model. This re-evaluation provides improved metrics of managerial efficiency, as the second stage has effectively removed environmental influences and statistical noise from the input data of DMUs. The primary advantage of this methodology lies in its capacity to eliminate the effects of non-managerial factor—namely, environmental influences and random noise—on efficiency, while preserving the managerial inefficiencies. This ensures that the final efficiency scores accurately reflect the internal managerial efficiency of the DMUs.

#### 3.1.1 First stage: Initial DEA efficiency evaluation.

In general, DEA models include the Charnes–Cooper–Rhodes (CCR) model and the Banker–Charnes–Cooper (BCC) model, with the former assuming constant returns to scale (CRS) [27], and the latter assuming variable returns to scale (VRS) [132–133]. The CCR model measures overall technical efficiency (TE), incorporating both scale efficiency (SE) and pure technical efficiency (PTE) simultaneously, with TE = SE × PTE, while the BCC model focuses solely on PTE evaluation.

In period *t* (*t* = 1, ⋯, *T*), there are *I* DMUs (*DMU*_*i*_^*t*^*, i =* 1, ⋯, *I*). Each DMU has *N* inputs and *M* outputs, where *x*^*t*^ and *y*^*t*^ represent the input and output vectors, respectively. These are defined as $x^{t}=(x_{1}^{t},x_{2}^{t},\cdots ,x_{N}^{t})\in R_{+}^{N}$, and $y^{t}=(y_{1}^{t},y_{2}^{t},\cdots ,y_{M}^{t})\in R_{+}^{M}$. The production possibility set for a given DMU during period *t* is defined as *T*^*t*^, and traditional DEA production technology is defined as follows:


$$\begin{array}{c} \;T^{t}=\left\{\begin{array}{c} \left(x^{t},y^{t}\right):\sum_{i=1}^{I}\lambda_{i}^{t}y_{im}^{t}\geq y_{im}^{t},m=1,\cdots ,M;\\ \sum_{i=1}^{I}\lambda_{i}^{t}x_{in}^{t}\leq x_{in}^{t},n=1,\cdots ,N;\lambda_{i}^{t}\geq 0 \end{array}\right\}\; \end{array}$$ (1)

In equation (1), *λ =* (*λ*_*1*_^*t*^, *λ*_*2*_^*t*^, ⋯, *λ*_*I*_^*t*^) represents the I-dimensional weight vector employed in assessing TE. Equation (1) demonstrates that traditional DEA measures production efficiency of DMUs based only on the production technology set *T*^*t*^ corresponding to the current period. Traditional DEA constructs the production technology set *T*^*t*^ using input-output data of cross-sectional DMUs for a single period. Consequently, when calculating the efficiency of DMUs across different periods (*t* = 1, 2, ⋯, *T*), distinct technological reference sets (*T* = *T*^*1*^, *T*^*2*^, ⋯, *T*^*T*^) are employed, which renders the efficiency values of DMUs across different periods non-comparable.

To address this limitation and enhance the comparability of efficiency across different periods, while also facilitating a longitudinal analysis of efficiency variations for each DMU, Pastor and Lovell [134] introduce a frontier construction method based on global benchmark technology, which forms the basis of global DEA. It utilizes panel data on inputs and outputs of all DMUs across all periods to establish a single production possibility set, serving as a common reference for production technology that encompasses all contemporaneous benchmark technologies [135]. Efficiency outcomes for different DMUs across various periods are then computed relative to this common frontier, thus forming a global DEA framework.

In this framework, *T*^*G*^ represents the global production technology set and *T*^*t*^ denotes the production technology set for each individual period. The construction of the global production technology set is as follows:


$$\;T^{G}=\cup_{t=1}^{T}T^{t}=T^{1}\cup T^{2}\cup \cdots \cup T^{T}$$ (2)

The linear programming formulation of the input-oriented global DEA method based on this global production technology set is presented below:


minθ     

$$s.t.\sum_{t=1}^{T}\sum_{i=1}^{I}\lambda_{i}^{t}x_{in}^{t}\leq {\theta x}_{n}^{t},\;n=1,\ldots ,N$$

$$\sum_{t=1}^{T}\sum_{i=1}^{I}\lambda_{i}^{t}y_{im}^{t}\geq y_{m}^{t\;}\;,\;m=1,\ldots ,M$$

$$\;\lambda_{i}^{t}\geq 0,\;i=1,\ldots ,I,\;t=1,\ldots ,T$$ (3)

Here, $\;\lambda_{i}^{t}$ denotes the weight variable. Equation (3) is based on CRS. When the constraint $\sum_{t=1}^{T}\sum_{i=1}^{I}\lambda_{i}^{t}=1$ is added, the model corresponds to VRS.

Another issue that needs clarification here is the number of DMUs. Generally, the number of DMUs should not be less than the product of the number of input and output indicators, nor should it be less than three times the sum of these indicators [136]. However, this is a rough guideline, and the actual number of DMUs should be assessed based on the results of the DEA analysis [137]. In this study, the number of DMUs appears to be 12 (provinces). However, when considering the time dimension (13 years) and the resulting panel data, the number of DMUs increases to 156 (12 × 13) in the global DEA model. Taking the technical efficiency of higher education input-output as an example, three sets of first-stage DEA results are presented in S7-S9 Tables. S7 Table matches the 12 provinces with the 13 years, numbering the matching results from 1 to 156. Therefore, the number of DMUs included in the model is 156, and TE values are calculated using the traditional DEA model. S8 Table uses the 12 provinces as DMUs and calculates TE values using the global benchmark technology (global DEA model). S9 Table also uses the 12 provinces as DMUs and calculates TE values using the traditional DEA model. It can be observed that the TE values in S7 Table and S8 Table are identical, and the distinction between the TE values is much greater than in S9 Table, indicating a more even distribution of TE values. Therefore, it can be concluded that in this study, the global DEA method is more robust and superior to the traditional DEA, and when global DEA is applied, the number of DMUs is expanded from 12 to 156 (provinces × years). Consequently, the final number of DMUs in this study is 156.

#### 3.1.2 Second stage: Utilizing SFA to decompose initial input slacks.

The second-stage analysis aims to disaggregate the input slacks from the first stage into three distinct components: environmental influences, managerial inefficiencies, and statistical noise resulting from measurement errors in data used to derive the initial slacks. Thus, in the second stage, each input slack is treated as a dependent variable, with *K* observable environmental variables *Z*_*i*_ serving as independent variables. For each input slack, an individual SFA regression equation is constructed, resulting in a total of *N* regression equations. By performing regression analysis, external environmental influences and stochastic factors are filtered out, isolating the input redundancies attributable solely to managerial inefficiencies for each DMU. The regression equation for the n-th input slack is as follows:


$$S_{ni}=f^{n}\left(Z_{i};\beta^{n}\right)+v_{ni}+u_{ni},\;n=1,2,...,N;i=1,2,...,I$$ (4)

where *S*_*ni*_ denotes the first-stage slack for the 𝑖-th DMU on the n-th input, representing the discrepancy between the optimal and actual input levels. *Z*_*i*_ denotes the environmental variables, where *Z*_*i*_ = [*Z*_1*i*_, ⋯, *Z*_*Ki*_]. *β*^*n*^ is the parameter vector associated with these environmental variables. *f*^*n*^*(Z*_*i*_;*β*^*n*^*)* represents the deterministic feasible slack frontiers, capturing the influence of environmental factors on *S*_*ni*_, and is typically defined as *f*^*n*^*(Z*_*i*_;*β*^*n*^*)* = *Z*_*i*_*β*^*n*^. However, this relationship contains noise. [*f*^*n*^*(Z*_*i*_;*β*^n^*)* + *v*_*ni*_] represents the stochastic feasible slack frontier, indicating the minimum achievable slack under the noise−influenced environment described by variables (*Z*_*i*_, *v*_*ni*_) and parameters (*β*_*n*_, *σ*_*un*_^2^). Any slack exceeding [*f*^*n*^*(Z*_*i*_;*β*^*n*^*) + v*_*ni*_] is attributed to managerial inefficiency. The composite error term (*v*_*ni* _+ *u*_*ni*_) comprises *v*_*ni*_, representing statistical noise, assumed to follow a normal distribution *v*_*ni *_~ *N*(0, *σ*_*vn*_^2^)*,* and *u*_*ni*_, representing managerial inefficiency, with *u*_*ni*_* *≥ 0, following a truncated normal distribution *u*_*ni*_ ~ *N*^*+*^(*μ*_*n*_, *σ*_*un*_^2^). The terms *v*_*ni*_, *u*_*ni*_ and *Z*_*i*_ are assumed to be mutually independent. Each of the *N* regressions in equation (4) can be estimated by maximum likelihood techniques.

Subsequently, the results of the regression analysis derived from the SFA model are employed to further adjust the input variables of the DMUs. This adjustment eliminates the influence of environmental and stochastic factors on efficiency, thereby aligning all DMUs within a common external environment. Below is the adjustment formula:


$$X_{ni}^{A}=X_{ni}+\left[\max_{i}\left\{Z_{i}{\hat{\beta }}^{n}\right\}-Z_{i}{\hat{\beta }}^{n}\right]+\left[\max\left\{{\hat{v}}_{ni}\right\}-{\hat{v}}_{ni}\right],$$

n=1,2,...,N;i=1,2,...,I (5)

Here, $X_{ni}^{A}$ and *X*_*ni*_ represent the adjusted inputs and pre-adjustment inputs, respectively, $\left[\max_{i}\left\{Z_{i}{\hat{\beta }}^{n}\right\}-Z_{i}{\hat{\beta }}^{n}\right]$ accounts for adjustments to environmental factors, $\max_{i}(Z_{i}{\hat{\beta }}^{n})$ signifies the adjustment of all DMUs to a standardized operating environment, specifically the least favorable environmental context identified within the sample. Meanwhile, $Z_{i}{\hat{\beta }}^{n}$ represents standardizing all DMUs to a uniform condition of “nature”, corresponding to the unluckiest situation observed in the dataset. Consequently, DMUs operating under relatively unfavorable environments and/or bad luck receive upward input adjustments of a smaller magnitude, while those in more favorable environments and/or enjoying good luck experience larger upward adjustments to their inputs. This ensures that all DMUs are adjusted to a uniform environmental level. $\left[\max\left\{{\hat{v}}_{ni}\right\}-{\hat{v}}_{ni}\right]$ addresses adjustments for stochastic error components, thus positioning all DMUs on an equivalent level of stochastic influence.

The SFA model is typically analyzed using Frontier 4.1 software, and the output file from the regression results contains estimated values for parameters such as *β*^*n*^, *σ*^*2*^ and *γ* corresponding to each slack variable, from which *σ*_*vn*_ and *σ*_*un*_ can be derived based on equation (6).


$$\sigma^{2}=\sigma_{vn}^{2}+\sigma_{\mu n}^{2},\;\gamma =\frac{\sigma_{un}^{2}}{\sigma_{vn}^{2}+\sigma_{un}^{2}}$$ (6)

The parameter *γ* represents the proportion of total variance attributable to managerial inefficiency variance.When *γ* approaches 1, the influence of managerial inefficiency predominates, while a *γ* value approaching 0 indicates that statistical noise is the dominant influence.

To implement equation (5), *v*_*ni*_ must be obtained for each DMU by separating statistical noise from managerial inefficiency in the residuals of equation (4). Following the methods proposed by Luo [138] and Kumbhakar and Lovell [139], the managerial inefficiency *u*_*ni*_ can be isolated, as expressed by the following equation:


$$\;E\left(u_{ni}|v_{ni}+u_{ni}\right)=\frac{\sigma \lambda }{1+\lambda^{2}}\left[\frac{\varphi \left(\frac{\varepsilon \lambda }{\sigma }\right)}{\Phi \left(\frac{\varepsilon \lambda }{\sigma }\right)}+\frac{\varepsilon \lambda }{\sigma }\right]\;$$ (7)

where σ_*_^2^* = σ*_*un*_^*2*^*σ*_*vn*_^*2*^*/ σ*^*2*^, *λ = σ*_*un*_*/ σ*_*vn*_, and *ε = v*_*ni *_*+ u*_*ni*_ is the composite error term. The function φ (·) and Ф (·) denote the density function and cumulative distribution function of the standard normal distribution, respectively.

After isolating the inefficiency component *u*_*ni*_, the random disturbance *v*_*ni*_ can be obtained, calculated as follows:


$$\;\hat{E}\left(v_{ni}|v_{ni}+u_{ni}\right)=S_{ni}-Z_{i}{\hat{\beta }}^{n}-\;\hat{E}\left(u_{ni}|v_{ni}+u_{ni}\right)$$

 n=1,2,...,N;i=1,2,...,I  (8)

Finally, by substituting *v*_*ni*_ into equation (5), the adjusted input variables are obtained.

#### 3.1.3 Third stage: Adjusted DEA efficiency evaluation.

In the third stage, DEA analysis is reapplied using the adjusted input variables. The specific steps are as follows: the adjusted input variable data $X_{ni}^{A}$, derived from the second stage, replace the original input variable data *X*_*ni*_, while output remains unchanged. Subsequently, the DEA model from the first stage is applied once again to calculate the efficiency scores. The efficiency values obtained for each DMU in this stage are thus purged of the effects of environmental factors and statistical noise, providing a more precise reflection of the true input-output efficiency.

### 3.2 Global Malmquist productivity index

Total Factor Productivity (TFP) is commonly used to assess the contribution of the overall efficiency of input factors to output growth. In the analysis of TFP changes, the Malmquist Total Factor Productivity Index (MI) is decomposed using the DEA method. The concept of the MI was originally introduced by Swedish economist and statistician Sten Malmquist in 1953, and it was subsequently developed by Caves, Färe, and others. Caves et al. [140] proposed employing the MI to compare the performance of a DMU with itself across different periods of time. Färe et al. [141] used DEA models to compute the MI, subsequently decomposing it into two principal components: technical efficiency change (EC) and technological change (TC). Since then, DEA-based MI has been extensively utilized for measuring changes in productivity. This approach employs a distance function to represent the production process with multiple inputs and outputs, thereby circumventing problems associated with subjective weight assumptions, since it is not dependent on a particular specification of the production function [140,142,143].

The MI for each DMU is defined as the ratio of its efficiency score at time *t* + 1 to that at time *t*, with both scores being computed relative to a designated benchmark technology [144]. However, when considering the production possibility set, the MI uses a contemporaneous production possibility set based on data observed within the same time period, which lacks circularity. Its adjacent period components can yield inconsistent assessments of productivity change. To address the limitations of non-circularity and disparate measurements, Pastor and Lovell [134] introduced a global Malmquist productivity index (GMI), where the production possibility set (or production frontier) is constructed using data from all DMUs across all periods. By adopting a common reference technology that spans all periods, the technical changes calculated between period *t* and *t* + 1 are benchmarked against the production technology of all periods, thereby ensuring the comparability of inter-temporal performance changes across the evaluated DMUs. This GMI satisfies circularity, offers a unified measure of productivity change, and is immune to the issue of infeasibility [19,134,145]. Moreover, technological change or frontier shift can be regarded as a global phenomenon, driven by factors such as changes in economic conditions or the introduction of new, more efficient technologies. In many cases, it is reasonable to assume that these factors are either uniform or, at the very least, highly analogous across all observations in an analysis. As a result, a single metric can be employed to represent the shift in the frontier for all DMUs [146]. Furthermore, the GMI expands the set of DMUs incorporated into the analysis, improving the precision of the frontier, particularly when the number of DMUs is limited. This enhancement increases the reliability and stability of results. In addition, by incorporating a global benchmark technology in the three-stage DEA model to construct the frontier, the use of the GMI becomes indispensable for accurately assessing productivity changes.

According to the definitions and formulas provided by Pastor and other scholars [134,147], consider a panel dataset with i= 1, ⋯, *I* DMUs observed over *t* = 1, ⋯, *T* time periods. DMUs utilize inputs $x\in R_{+}^{N}$ *t*o generate outputs $y\in R_{+}^{M}$. The production technology at time *t* is represented by $T^{t}=\left\{(x^{t},y^{t})|x^{t}can\;produce\;y^{t}\right\}$.

A global Malmquist productivity index is defined on $T^{G}$as:


$$M^{G}\left(x^{t},y^{t},x^{t+1},y^{t+1}\right)=\frac{D^{G}(x^{t+1},y^{t+1})}{D^{G}(x^{t},y^{t})}$$ (9)

Here, the output distance functions $D^{G}(x,y)=min\left\{\emptyset >0|(x,y/\emptyset )\in T^{G}\right\}$. *D*^*G*^ (*x*^*t+1*^, *y*^*t+1*^) represents the DEA efficiency score of (*x*^*t+1*^, *y*^*t+1*^) when benchmarked against the global frontier, and *D*^*G*^ (*x*^*t*^, *y*^*t*^) denotes the DEA efficiency score of (*x*^*t*^, *y*^*t*^), similarly benchmarked against the global frontier. Since the GMI employs a single global benchmark technology, constructed from all periods as the reference frontier for all sample observations, the calculation does not require the use of a geometric mean [134,148].

By further decomposing the equation (9), the GMI can be separated into the EC and TC indices.


$$M^{G}\left(x^{t},y^{t},x^{t+1},y^{t+1}\right)=\frac{D^{t+1}(x^{t+1},y^{t+1})}{D^{t}(x^{t},y^{t})}\times \left[\frac{D^{G}(x^{t+1},y^{t+1})}{D^{G}(x^{t},y^{t})}\times \frac{D^{t}(x^{t},y^{t})}{D^{t+1}(x^{t+1},y^{t+1})}\right]$$ (10)

$$EC=\;\frac{D^{t+1}(x^{t+1},y^{t+1})}{D^{t}(x^{t},y^{t})}$$ (11)

$$TC=\;\frac{D^{G}(x^{t+1},y^{t+1})}{D^{G}(x^{t},y^{t})}\times \frac{D^{t}(x^{t},y^{t})}{D^{t+1}(x^{t+1},y^{t+1})}=\frac{\frac{D^{G}(x^{t+1},y^{t+1})}{D^{t+1}(x^{t+1},y^{t+1})}}{\frac{D^{G}(x^{t},y^{t})}{D^{t}(x^{t},y^{t})}}$$ (12)

Specifically, *D*^*G*^(*x*^*t+1*^, *y*^*t+1*^)/*D*^*t+1*^(*x*^*t+1*^, *y*^*t+1*^) quantifies the distance between the period *t* + 1 frontier and the global frontier, where a greater ratio denotes a closer alignment between the *t* + 1 period frontier and the global frontier. Similarly, *D*^*G*^(*x*^*t*^, *y*^*t*^)/*D*^*t*^(*x*^*t*^, *y*^*t*^) represents the distance between the frontier at period *t* and the global frontier, with a higher ratio suggesting greater proximity of the period *t* frontier to the global frontier. The ratio of these two terms captures the relative shift of the period *t* + 1 frontier compared to the period *t* frontier. TC refers to the advancement of production technology, which shifts the production frontier outward, allowing for higher output with the same input levels, and reflects impacts relevant to the higher education system as a whole. This suggests that technological progress plays a significant role in improving production efficiency. EC captures variations in the relative efficiency of a DMU, reflecting the extent to which a DMU approaches the production frontier, and it can be further disaggregated into two components: pure technical efficiency change (PEC) and scale efficiency change (SEC), as expressed below:


$$EC\;\left(x^{t},y^{t},x^{t+1},y^{t+1}\right)=PEC\;\left(x^{t},y^{t},x^{t+1},y^{t+1}\right)\times SEC\;\left(x^{t},y^{t},x^{t+1},y^{t+1}\right)$$ (13)

GMI is finally expressed as GMI = PEC×SEC×TC. When the GMI > 1, total factor productivity improves, indicating increased output efficiency under the same input conditions. when the GMI < 1, total factor productivity declines, reflecting reduced production efficiency. When GM = 1, TFP remains constant.

### 3.3 Tobit model

The Tobit model is specifically developed to address the issue of censored dependent variables. Parameters in this model are estimated through maximum likelihood estimation, enabling it to effectively handle observations where efficiency scores are either 0 or 1, thereby ensuring the robustness and consistency of the estimation results. In this study, the dependent variable is the DEA efficiency score, adjusted in the third stage, ranging between (0, 1]. Employing the ordinary least squares method for parameter estimation may lead to distortions in the results [149–150]. Given the bounded nature of the dependent variable, the Tobit model is used to address the censoring of efficiency scores, allowing for precise estimation of factors influencing efficiency and ensuring unbiased, consistent estimates. It is assumed that the latent efficiency score *y*_it_^*^ is an unrestricted continuous variable, while the observed efficiency score *y*_*it*_ is a truncated variable. The Tobit model is represented in the following form [151]:


$$y_{it}^{*}=x_{it}\beta +\varepsilon_{it}\;$$

$$\begin{array}{c} y_{it}=\left\{ \begin{array}{c} 0,if\;y_{it}^{*}\leq 0\\ y_{it}^{*},\;if\;0<y_{it}^{*}\leq 1 \end{array}\right.\; \end{array}$$ (14)

where *x*_*it*_ represents the vector of independent variables, encompassing various factors influencing efficiency; *β* denotes the regression coefficients to be estimated; and *ε*_*i*_ is the error term, with *ε*_*i*_ ~ *N* (0, *σ*^*2*^).

## 4. Variables selection and data collection

### 4.1. Input–output variables

Following the overarching theoretical framework for efficiency measurement, this study develops an evaluation index system for the input-output efficiency of higher education from both input and output dimensions. Widely recognized in academia, the input indicators encompass financial, human, and physical resource inputs [152], while the output indicators are primarily structured around the three core functions of higher education: talent cultivation, research outcomes, and social service [88]. Building on prior research [87,119,153–155], as detailed in S1 Table, this study develops a comprehensive evaluation index system for higher education input-output efficiency, comprising seven input and seven output indicators, as presented in Table 1.

**Table 1:** Indicators of Input-Output in Higher Education.

Indicator Type	Actual Variable	Variable Code	Unit
Financial Input	General Public Budget Education Expenditure of HEIs	X_1_	Thousand CNY
	Intramural Expenditures on R&D in Higher Education	X_2_	Ten Thousand CNY
Human Input	Number of Full-time Faculty in HEIs	X_3_	Person
	Full-time Equivalent of R&D Personnel in HEIs	X_4_	Man-Year
Physical Input	Number of HEIs	X_5_	Institution
	Campus Area of HEIs	X_6_	Square Meters
	Total Fixed Assets Value of HEIs	X_7_	Ten Thousand CNY
Talent Cultivation	Number of Regular Undergraduate Graduates (or Completers) from HEIs	Y_1_	Person
	Number of Master’s Graduates (or Completers) from HEIs	Y_2_	Person
	Number of Doctoral Graduates (or Completers) from HEIs	Y_3_	Person
Scientific Research	Number of Academic Papers Published by HEIs	Y_4_	Piece
	Number of Scientific Books Published by HEIs	Y_5_	Kind
Social Services	Number of Patent Applications by HEIs	Y_6_	Piece
	Number of Technology Transfer Contracts by HEIs	Y_7_	Item

#### 4.1.1 Input variables.

Firstly, financial input. Financial input serves as the economic foundation for the long-term and steady advancement of higher education, encompassing government appropriations, tuition revenue, and self-raised funds. However, due to incomplete data on self-raised funds and tuition revenue, this study, drawing on existing research, selects general public budget education expenditure of HEIs and intramural expenditures on R&D in higher education as the primary indicators of financial input. General public budget education expenditure of HEIs refers to the regular financial resources provided by the government, characterized by stability and sustainability. The funding primarily supports teaching, infrastructure development, faculty salaries, and daily operations. As the core funding source for higher education, it directly impacts quality enhancement, facility upgrades, and faculty development. Reflecting the extent of governmental support, general public budget education expenditure of HEIs serves as a critical financial indicator for assessing the input-output efficiency of higher education. Intramural expenditures on R&D in higher education represents the internal funds allocated by universities for research and development activities, sourced mainly from institutional budgets or research project grants. These funds are a vital driver of technological innovation and development within HEIs. Effective allocation and utilization of these funds facilitate the commercialization of research outcome, foster university-industry collaboration, and reflect the management efficiency of HEIs within the innovation ecosystem. Together, these two financial input indicators form the foundation for assessing the input-output efficiency in higher education, providing a comprehensive perspective of the financial investment in both educational and research domains and their overall effectiveness.

Secondly, human resource input. Human resource input is a critical factor driving teaching and research activities in higher education, primarily encompassing the number of full-time faculty in HEIs and the full-time equivalent of R&D personnel in HEIs. The former represents the core strength in talent cultivation within universities; both the quantity and quality of faculty directly impact teaching quality and research capacity, ultimately determining the effectiveness of talent development. The latter is a standardized measure of the actual time invested by research personnel, converting the time spent by all individuals engaged in R&D activities into equivalent full-time labor. This measure quantifies the intensity of human resource input in university research activities and effectively assesses the actual commitment of research staff, reflecting the level of labor investment in the research domain. Together, these two human resource input indicators effectively quantify the investment in human capital within HEIs, providing a comprehensive reflection of the intensity and quality of key inputs in teaching quality, research capacity, and human capital development.

Thirdly, physical resource input. Physical resource input represents the foundational conditions for the development of higher education, encompassing long-term, valuable stock assets and other resources. This study selects the number of HEIs, the campus area of HEIs, and the total fixed asset value of HEIs as key indicators. The number of HEIs reflects the distribution and density of educational establishments within a specific region, representing the carrying capacity of regional educational resources and serving as a crucial indicator of infrastructure expansion in higher education within that region. The campus area of HEIs measures the physical space resources of institutions, reflecting the basic conditions provided for teaching, research, and campus life. Adequate physical space contributes to improved educational quality and research capacity. The total fixed asset value of HEIs represents the total value of material resources within institutions, including land, buildings, educational equipment, and research facilities, and serves as an important economic indicator of physical resource investment levels. These three indicators collectively assess the physical resource input in higher education, providing a critical material foundation for the outputs achieved by HEIs.

#### 4.1.2 Output variables.

Firstly, talent cultivation. A core function of higher education is talent cultivation, with the training of high-quality graduates serving as a direct manifestation of educational outcomes in HEIs and as a significant means of contributing to society. In terms of educational levels, the output of HEIs is primarily reflected in the training of undergraduates, master’s students, and doctoral students. Thus, this study selects the number of regular undergraduate graduates, master’s graduates, and doctoral graduates as key indicators for measuring talent cultivation outcomes. The number of general undergraduate graduates constitutes the majority of university graduates, who are the primary focus of universities’ educational resource investment and also constitute the foundational talent supply across various sectors of society. Consequently, the number of undergraduate graduates reflects the output efficiency of universities in large-scale talent cultivation. The number of master’s graduates indicates the output capacity of HEIs in high-level talent cultivation, serving as an important indicator of teaching quality and the integration of research and education. The number of doctoral graduates is a critical measure of a HEI’s capacity for the highest level of talent cultivation and research strength, directly reflecting its ability to produce innovative talent.

Secondly, research output. It is a significant outcome of higher education, typically encompassing three primary dimensions: basic research, applied research, and experimental development. Basic research serves as the cornerstone of academic activities in higher education, aimed at advancing the frontiers of knowledge and establishing a theoretical basis for subsequent research endeavors. In contrast, applied research and experimental development focus on translating the findings of basic research into practical applications, addressing societal needs, and driving technological progress. Therefore, this study selects the two most representative indicators to measure basic research output: the number of published academic papers by HEIs and the number of scientific books by HEIs. Academic papers reflect HEIs’ performance in research innovation and scholarly contributions, while scientific publications demonstrate the systematic synthesis of research results and academic impact. As for the outputs of applied research and experimental development, these are reflected through the dimension of social service, evidenced by tangible outcomes such as technology transfers and patent applications.

Thirdly, social service. Social service represents a key function of higher education in fostering socio-economic progress, with patents and their subsequent commercialization and industrialization serving as direct reflections of HEIs’ contributions to society. For the purpose of measuring the output level of higher education in social service, this study selects the number of patent applications by HEIs and the number of technology transfer contracts by HEIs as core indicators. The number of patent applications reflects HEIs’ capacity for technological innovation and intellectual property protection, which is a prerequisite for the conversion of research outcomes into practical applications and serves as a measure of how well university research aligns with societal needs. The number of technology transfer contracts, conversely, indicates the actual industrialization level of university research outcomes, demonstrating the depth and breadth of university-industry collaboration. Together, these two indicators effectively represent HEIs’ performance in intellectual property creation and the transformation of scientific achievements, providing a comprehensive view of the role and influence of higher education in serving society.

### 4.2 External environmental variables

In the second phase of the three-stage DEA, to obtain more accurate and true efficiency values, an SFA regression on input slack is required to eliminate the effects of external environmental factors and statistical noise on the efficiency scores obtained in the first stage. According to the model of Battese and Coelli [156–157], these environmental variables denote external factors that impact SFA technical efficiency and are incorporated into the variance of the SFA technical inefficiency distribution. Therefore, for the external environmental factors entered into the SFA regression model, this study selects those variables that significantly impact input slack in higher education, specifically: per capita Gross Regional Product (GRP), the proportion of government fiscal expenditure on education, urbanization rate, density of highways and railways, and the proportion of the tertiary industry in GRP. These variables comprehensively assess the influence of the external environment on input slack (managerial inefficiency) in higher education from the perspectives of macroeconomic conditions, government investment, urbanization, infrastructure, and labor market demand.

#### 4.2.1 Per capita GRP.

The economic development status of the region where HEIs are situated serves as a critical macroeconomic framework, significantly influencing resource allocation and operational dynamics within these institutions. The GDP level partially reflects the extent of financial support that the local economy provides to the development of higher education [29]. Economically advanced regions typically possess more abundant fiscal resources and governmental support, creating a favorable research environment and well-developed infrastructure for HEIs. This, in turn, optimizes resource allocation, directly enhancing research output and teaching quality [158]. Additionally, a higher GDP level attracts more investment to universities, further boosting research and teaching efficiency. In numerous studies, per capita GDP is commonly employed as a key indicator to reflect a region’s economic development level and its potential influence on educational investment [55].

#### 4.2.2 Proportion of government fiscal expenditure on education.

The proportion of government fiscal expenditure on education not only directly impacts infrastructure development and research investment in HEIs but also plays a pivotal role in the enhancement of research facilities, talent acquisition, and support for research projects. It is one of the primary factors influencing research output and teaching quality in universities [159]. Particularly in contexts where regional disparities in higher education development are pronounced, government fiscal support is regarded as an effective means of narrowing regional gaps, promoting equity, and improving the quality of higher education. In economically underdeveloped areas, government financial support serves as the main driving force for enhancing the output efficiency of higher education. This factor also reflects the educational policy orientation of national or local governments, reinforcing its critical role in the allocation of higher education resources.

#### 4.2.3 Urbanization rate.

Urbanization rate provides a clear reflection of the level of regional infrastructure and the capacity to aggregate educational resources [160–162]. As urbanization progresses, substantial human and material resources concentrate in urban areas, aiding in the optimized allocation of higher education resources. Furthermore, the urbanization rate serves as a key indicator of socio-economic development, profoundly influencing the flow and concentration of talent. Higher urbanization rates are typically associated with a higher-quality educational environment and better research conditions, pooling abundant social and economic resources to foster the development of higher education. This, in turn, enhances the efficiency of talent cultivation, research, and teaching in HEIs.

#### 4.2.4 Density of highways and railways.

The construction and enhancement of transportation networks not only facilitate the flow of resources within and across regions but also significantly strengthen collaborative efficiency among HEIs. High-density transportation infrastructure supports the effective movement of educational resources—such as faculty, students, and research equipment—across regions, reducing the spatial and temporal distances between institutions and thereby improving resource allocation efficiency. Existing studies indicate that transportation infrastructure development has a substantial positive impact on regional resource mobility and economic efficiency [163]. For geographically remote areas, such as western China, improved transportation conditions are particularly significant for the advancement of regional higher education. Enhanced transportation can effectively alleviate the limitations on HEIs’ access to external resources, promote talent mobility, and further boost the overall output efficiency of higher education.

#### 4.2.5 Proportion of tertiary industry in GRP.

The increasing proportion of the tertiary industry in GRP reflects both the quality of economic development and the market demand for highly educated talent, serving as an effective measure of the market’s capacity to absorb higher education graduates and exerting a significant influence on the multidimensional investment in higher education resources. Firstly, the expansion of the tertiary sector is often associated with the growth of technology- and knowledge-intensive industries, which increasingly demand highly skilled talent. This demand drives the government and society to intensify investments in higher education to cultivate talent aligned with industry needs. Secondly, the development of the tertiary industry strengthens regional economic capacity, enhancing local government fiscal capabilities and thereby increasing financial support for higher education. Lastly, as the tertiary sector’s demand for highly qualified labor grows, more talent is drawn to the education field, promoting improvements in faculty development and teaching resources within higher education. These factors interact to collectively reinforce the overall investment level in higher education. Existing research indicates that the proportion of the added value of the tertiary industry in GDP exerts a significantly positive influence on the effective allocation of higher education resources [16].

### 4.3 Determinant variables in the Tobit model

In Section 2.3, “Factors influencing higher education efficiency”, a systematic review of the factors affecting input-output efficiency in higher education has been conducted. Grounded in the theoretical foundations of existing literature and considering the unique context of western China, this study constructs an analytical framework from three dimensions: the regional socio-economic environment, human capital, and educational funding.

In the dimension of the regional socio-economic environment, the relatively slow economic development in western China means that local government fiscal revenue directly impacts its capacity to support higher education. Therefore, the proportion of regional fiscal revenue to gross regional product (RFR/GRP) is selected as an indicator to assess the level of local economic development and its support for higher education.

In the dimension of human capital, given the relative lag in the recruitment and development of high-level talent within western HEIs, the structure of academic titles serves as a direct indicator of the quality of internal human capital. Accordingly, this study selects the proportion of full-time faculty with senior and associate senior academic titles (SAS/FTF) as a representative indicator of internal human capital level in HEIs, reflecting the overall academic and research capacity of the faculty. At the same time, the proportion of employees with college education or above (PECE) is used to assess the overall level of external human capital in western China, indicating the capacity of the regional education system to supply qualified labor to the workforce.

In the dimension of educational funding, per-student education expenditure (PSEE) serves as a key indicator of regional educational resource investment intensity, directly reflecting the allocation of resources. This metric is particularly significant in western China, where economic underdevelopment and uneven resource distribution prevail. For HEIs in the region, which operate under relatively constrained resources, efficient allocation of funding is essential for improving institutional efficiency. Within this context, the proportion of higher education operating expenses to total education expenditure (HEOE/TEE) further reveals the structural characteristics of funding allocation, assessing the efficiency of financial resources directed toward routine teaching and research activities.

### 4.4 Data description

The study spans from 2010 to 2022 and includes 12 provinces, municipalities, and autonomous regions in China’s western region, specifically: Chongqing Municipality, Sichuan, Yunnan, Guizhou, Shaanxi, Gansu, Qinghai, Guangxi Zhuang Autonomous Region, Tibet Autonomous Region, Ningxia Hui Autonomous Region, Xinjiang Uygur Autonomous Region, and Inner Mongolia Autonomous Region. Data on higher education input and output variables are sourced from *Educational Statistics Yearbook of China*, *China Statistical Yearbook on Science and Technology*, *China Educational Finance Statistical Yearbook*, and *Compilation of Science and Technology Statistics of Higher Education Institutions*, spanning from 2010 to 2023. Specifically, data for *X*_*3*_, *X*_*6*_, *X*_*7*_, *Y*_*1*_, *Y*_*2*_, and *Y*_*3*_ are derived from the *Educational Statistics Yearbook of China*, *X*_*2*_, *X*_*4*_, *X*_*5*_, *Y*_*5*_, and *Y*_*6*_ from the *China Statistical Yearbook on Science and Technology, Y*_*4*_ and *Y*_*7*_ from the *Compilation of Science and Technology Statistics of Higher Education Institutions*, and *X*_*1*_ from the *China Educational Finance Statistical Yearbook*.

The data for external environmental variables and influencing factors are sourced from various yearbooks spanning from 2010 to 2023. Regional fiscal revenue, GRP, per capita GRP, urbanization rate, proportion of tertiary industry in GRP, and highway and railway mileage are obtained from the *China Statistical Yearbook*; data on the proportion of government fiscal expenditure on education and the proportion of regional fiscal revenue to GRP come from the *Finance Yearbook of China*; per-student education expenditure and the proportion of higher education operating expenses to total education expenditure are sourced from *China Educational Finance Statistical Yearbook*; the proportion of full-time faculty with senior and associate senior academic titles is obtained from the *Educational Statistics Yearbook of China*; and the proportion of employees with college education or above is derived from the *China Labor Statistical Yearbook*.

To obtain a deeper insight into the distributional characteristics and fundamental statistical properties of the higher education input and output variables, this study conducts descriptive statistical analysis on seven input variables(*X*_*1*_–*X*_*7*_) and seven output variables (*Y*_*1*_–*Y*_*7*_). The analysis includes sample size, mean, standard deviation, minimum, maximum, and coefficient of variation. The descriptive statistics for each relevant variable are presented in Table 2.

**Table 2:** Descriptive Statistics of Higher Education Input-output Variables.

Variable	Count	Mean	SD	Min	Max	Median	CV
**X**_**1**_	156	9.94 × 10^6^	7.41 × 10^6^	5.51 × 10^5^	3.73 × 10^7^	8.54 × 10^6^	0.75
**X**_**2**_	156	1.80 × 10^5^	2.54 × 10^5^	2577	1.92 × 10^6^	70532	1.41
**X**_**3**_	156	33561.71	24591.12	2195	1.05 × 10^5^	29089.5	0.73
**X**_**4**_	156	7470.58	7476.31	253	34322	4737.5	1.00
**X**_**5**_	156	56.56	32.47	5	134	55.5	0.57
**X**_**6**_	156	3.94 × 10^7^	2.57 × 10^7^	2.92 × 10^6^	1.03 × 10^8^	3.86 × 10^7^	0.65
**X**_**7**_	156	3.93 × 10^6^	3.41 × 10^6^	1.37 × 10^5^	1.59 × 10^7^	3.08 × 10^6^	0.87
**Y**_**1**_	156	71782.21	56855.845	4553	2.53 × 10^5^	63182	0.79
**Y**_**2**_	156	9557.97	9010.84	161	41553	7141.5	0.94
**Y**_**3**_	156	646.72	872.22	1	3335	214	1.35
**Y**_**4**_	156	17062.96	17033.061	347	78687	10940.5	1.00
**Y**_**5**_	156	713.29	573.07	22	2012	647	0.80
**Y**_**6**_	156	3490.79	4696.08	0	20240	1576.5	1.35
**Y**_**7**_	156	214.79	402.41	0	1943	21	1.87

## 5. Results and discussion

### 5.1 Empirical results of three-stage DEA

#### 5.1.1 First stage: Input-oriented VRS DEA model results.

To begin with, without accounting for environmental and random factors, a DEA model was constructed based on the selected input and output indicators, using a global benchmark technology to establish the production frontier. This model enabled an initial analysis of the input-output efficiency of higher education across 12 provinces in western China from 2010 to 2022. The model was solved using MaxDEA software, and the results are presented in Table 3.

**Table 3:** Initial Technical Efficiency of Higher Education in Western China (2010-2022).

Province	2010	2011	2012	2013	2014	2015	2016	2017	2018	2019	2020	2021	2022	Mean	Rank
**Chongqing**	1.0000	1.0000	1.0000	1.0000	1.0000	1.0000	1.0000	1.0000	1.0000	1.0000	1.0000	1.0000	1.0000	1.0000	1
**Sichuan**	1.0000	1.0000	0.9878	0.9633	1.0000	1.0000	0.9626	0.9792	0.9952	0.9725	0.9688	0.9527	1.0000	0.9832	4
**Yunnan**	1.0000	1.0000	1.0000	0.9423	0.9458	1.0000	1.0000	1.0000	1.0000	0.9428	0.9962	1.0000	1.0000	0.9867	3
**Guizhou**	1.0000	0.9129	0.8959	0.7914	0.7935	0.8679	0.8647	0.9564	0.8656	0.7292	0.7967	0.8018	0.8385	0.8550	12
**Guangxi**	1.0000	1.0000	1.0000	1.0000	1.0000	0.9854	0.9662	0.9036	0.8250	0.8539	0.9987	0.8534	1.0000	0.9528	8
**Tibet**	1.0000	0.9236	1.0000	0.9242	0.9178	0.8144	1.0000	0.9073	0.8493	0.8596	0.8745	0.8173	0.8188	0.9005	10
**Shaanxi**	1.0000	1.0000	1.0000	1.0000	1.0000	1.0000	1.0000	1.0000	1.0000	1.0000	1.0000	1.0000	1.0000	1.0000	1
**Gansu**	1.0000	1.0000	1.0000	1.0000	1.0000	1.0000	1.0000	1.0000	0.9112	0.8613	0.9599	0.9706	1.0000	0.9772	6
**Ningxia**	1.0000	1.0000	1.0000	1.0000	0.8971	0.8445	0.8549	0.9566	0.8168	0.8046	0.7625	0.7426	0.8362	0.8858	11
**Qinghai**	1.0000	1.0000	1.0000	1.0000	0.8448	0.8261	0.9004	0.7712	0.8404	0.9328	1.0000	0.9170	1.0000	0.9256	9
**Xinjiang**	1.0000	1.0000	0.9847	0.9862	0.9912	0.9982	0.9303	0.9473	0.9247	1.0000	1.0000	1.0000	1.0000	0.9817	5
**Inner Mongolia**	1.0000	0.9542	0.9562	0.9793	1.0000	1.0000	1.0000	0.9984	0.9101	0.8908	0.9421	0.9194	1.0000	0.9654	7

Technical efficiency reflects the overall efficiency level of each DMU given the established resource inputs. Based on the average technical efficiency scores of the 12 provinces in western China from 2010 to 2022, Chongqing and Shaanxi rank first, each with an average technical efficiency score of 1, signifying DEA-efficient units. Additionally, both Chongqing and Shaanxi show zero slack variables, indicating no redundant inputs or insufficient outputs, which suggests that higher education input-output in these two provinces has achieved strong DEA efficiency, maximizing output under current input conditions. Following them are Yunnan (0.9867) and Sichuan (0.9832), both approaching DEA-efficient levels. Provinces with lower technical efficiency include Guizhou (0.8550), Ningxia (0.8858), and Tibet (0.9005), indicating certain deficiencies in the allocation and management of higher education resources in these regions.

Overall, the input-output technical efficiency of higher education in western China is relatively favorable, with high technical efficiency observed in Shaanxi, Chongqing, Yunnan, and Sichuan. However, there exists an imbalance in input-output technical efficiency across provinces, with Guizhou, Ningxia, and Tibet exhibiting lower levels of efficiency. Notwithstanding this disparity, the difference between the provinces with highest and lowest average technical efficiency scores is not particularly pronounced, with the maximum difference being 0.145 points.

#### 5.1.2 Second stage: SFA regression results.

The slack variables of the seven input variables selected in the first stage of measurement were used as dependent variables, while five environmental variables—per capita GRP, the proportion of government fiscal expenditure on education, urbanization rate, density of highways and railways, and the proportion of tertiary industry in GRP—served as independent variables. Regression analysis was conducted using Frontier 4.1 software, with results shown in Table 4.

**Table 4:** SFA Regression Results.

Variables	Slack in General Public Budget Education Expenditure of HEIs	Slack in Intramural Expenditures on R&D in Higher Education	Slack in Number of Full-time Faculty in HEIs	Slack in Full-time Equivalent of R&D Personnel in HEIs	Slack in Number of HEIs	Slack in Campus Area of HEIs	Slack in Total Fixed Assets Value of HEIs
**Constant**	−6.15E + 06***	−4.46E + 05***	−9.56E + 03***	−9.44E + 03***	−1.72E + 01***	−2.28E + 07***	−2.14E + 06***
**Per Capita GRP**	−8.36E + 00***	9.78E-01***	−2.17E-02	−3.12E-06	−9.62E-05***	−3.58E + 01***	1.10E + 00***
**Government Expenditure**	1.36E + 05***	8.92E + 03***	2.08E + 02***	8.56E + 01***	4.66E-01***	6.25E + 05***	3.90E + 04***
**Urbanization Rate**	2.89E + 04***	1.56E + 03***	5.87E + 01**	7.52E + 01***	1.34E-01***	8.39E + 04***	1.01E + 04***
**Density**	−2.15E + 05***	−2.29E + 04***	−2.22E + 02	−1.01E + 03***	1.42E + 00***	−4.69E + 05***	−1.03E + 05***
**Tertiary Industry**	5.41E + 04***	2.82E + 03***	7.87E + 01***	7.41E + 01***	9.17E-02***	1.84E + 05***	1.68E + 04***
**σ** ^**2**^	2.46E + 12***	4.00E + 10***	9.51E + 06***	1.58E + 07***	8.44E + 01***	4.32E + 13***	4.30E + 11***
**γ**	9.88E-01***	1.00E + 00***	9.93E-01***	1.00E + 00***	1.00E + 00***	9.90E-01***	1.00E + 00***
**Log**	−2.34E + 03***	−1.99E + 03***	−1.36E + 03***	−1.38E + 03***	−4.45E + 02***	−2.56E + 03***	−2.19E + 03***
**LR**	6.96E + 01***	1.43E + 02***	7.86E + 01***	1.23E + 02***	1.14E + 02***	7.26E + 01***	9.33E + 01***

Note: *, **, *** indicate significance at the 10%, 5%, and 1% statistical levels, respectively, based on t-test values.

The one-sided generalized likelihood ratio (LR) statistic has a critical value of 14.325 at the 1% significance level. As presented in the Table 4, the LR values for all seven models exceed 14.325, indicating that each of the seven slack variables passed the 1% significance test, thereby rejecting the null hypothesis of no managerial inefficiency. This suggests that managerial inefficiency significantly impacts each of the seven input variables, confirming the presence of efficiency losses in the input-output process of higher education in western China. Therefore, it is both reasonable and essential to further examine the relationship between external environmental factors and higher education input-output efficiency by constructing a SFA model.

In the regression results, the γ values are all greater than 0.9, indicating that managerial inefficiency plays a dominant role in input-output efficiency. The input slack variables represent the amount of input that could be reduced by improving management levels. In the regression model, a negative regression coefficient implies that an increase in the value of an environmental variable helps reduce input slack, thereby decreasing input waste and enhancing resource utilization efficiency. On the other hand, a positive regression coefficient indicates that an increase in the environmental variable raises input slack, leading to input waste or reduced output, thus impeding effective resource utilization. The subsequent section presents an analysis of the impact of each environmental variable on input slack.

Per capita GRP has a significant negative impact on the slack in general public budget education expenditure of HEIs, the number of HEIs, and campus area, indicating that an increase in per capita GRP helps reduce redundancies in these input resources. However, it exerts a significant positive impact on the slack in intramural expenditures on R&D in higher education and the total fixed assets value of HEIs, implying that an increase in per capita GRP leads to waste in these types of inputs. Thus, while growth in per capita GRP can optimize the allocation of certain educational resources, it may also exacerbate redundancy in others.

The proportion of government fiscal expenditure on education, the urbanization rate, and the proportion of tertiary industry in GRP all have a significant positive effect on the slack of all input variables. This indicates that increased government expenditure on education, a higher level of urbanization, and the development of the tertiary industry exacerbate the waste of higher education resources, leading to relatively reduced educational output.

The increase in highway and railway density has a significant impact on the allocation of higher education resources, particularly by enhancing the accessibility of the transportation network and reducing the distance between remote or underdeveloped regions and other areas. As shown in Table 4, improvements in transportation infrastructure have a significant negative effect on several higher education input slacks, notably reducing redundancy in general public budget education expenditure, intramural expenditures on R&D, full-time equivalent of R&D personnel, campus area, and total fixed assets value of HEIs. This suggests that enhanced transportation accessibility improves resource mobility and personnel flow, optimizing the allocation of higher education resources and reducing unnecessary input waste. However, an increase in highway and railway density has a significant positive impact on the slack in the number of HEIs. Although improved transportation fosters regional economic development and population mobility, potentially leading to the establishment of more institutions within the region, it does not necessarily guarantee the effective utilization of these new institutions. Population mobility and uneven distribution in western China may result in a mismatch between the increase in the number of HEIs and actual educational demand, thereby exacerbating redundancy in institutional numbers.

The above analysis indicates that various environmental variables have significant impacts on input slack in higher education, though their mechanisms of influence differ, resulting in varying degrees of redundancy across different types of input variables. This variation can affect the scientific validity of input-output efficiency results. This result aligns with the findings of Wu et al. [29], whose study on the efficiency of HEIs across 31 provinces in China similarly concluded that environmental variables and stochastic noise exerted a significant influence on the performance of DMUs. Therefore, when measuring the input-output efficiency of higher education, it is imperative to adjust input variables by accounting for the impact of external environmental and stochastic factors, ensuring that all provinces in western China face the same environmental and random conditions. Recalculating higher education input-output efficiency under these standardized conditions helps to ensure the fairness and accuracy of the efficiency measurements.

#### 5.1.3 Third stage: Efficiency adjusted for environmental influences and random noise.

According to the regression results from the second phase, the original input variables were scientifically and systematically adjusted, eliminating the impacts of external environmental factors and random noise. The adjusted input variables, along with the original output variables were then defined as the input and output variables for the third phase. These variables were re-evaluated using the DEA model from the first phase to obtain the adjusted efficiency values for higher education input-output in western China. This adjusted efficiency value more accurately reflects the true input-output efficiency of higher education in this region.

(1) Analysis of provincial average technical efficiency.

Table 5 presents the technical efficiency values of each province in western China from 2010 to 2022.

**Table 5:** Adjusted Technical Efficiency of Higher Education in Western China (2010-2022).

Province	2010	2011	2012	2013	2014	2015	2016	2017	2018	2019	2020	2021	2022	Mean	Rank
**Chongqing**	1.0000	0.9429	1.0000	0.9863	1.0000	1.0000	1.0000	1.0000	1.0000	1.0000	1.0000	1.0000	1.0000	0.9946	2
**Sichuan**	1.0000	1.0000	1.0000	0.9881	1.0000	1.0000	0.9843	1.0000	1.0000	0.9848	0.9895	0.9599	1.0000	0.9928	3
**Yunnan**	1.0000	0.9759	1.0000	0.8341	0.9093	1.0000	1.0000	1.0000	1.0000	0.9692	1.0000	1.0000	1.0000	0.9760	5
**Guizhou**	1.0000	0.9010	0.8608	0.7797	0.7837	0.8815	0.8712	0.8436	0.8006	0.7260	0.8121	0.8342	0.8462	0.8416	9
**Guangxi**	1.0000	0.9721	1.0000	0.9956	1.0000	0.9973	0.8671	0.9301	0.8699	0.9099	1.0000	0.8802	1.0000	0.9556	6
**Tibet**	0.3501	0.3153	0.3532	0.3223	0.3552	0.3728	0.3663	0.4322	0.3791	0.4321	0.4044	0.4408	0.4532	0.3828	12
**Shaanxi**	1.0000	1.0000	1.0000	1.0000	1.0000	1.0000	1.0000	1.0000	1.0000	1.0000	1.0000	1.0000	1.0000	1.0000	1
**Gansu**	0.9826	0.9462	1.0000	1.0000	1.0000	1.0000	1.0000	1.0000	0.9358	0.8703	0.9757	0.9929	1.0000	0.9772	4
**Ningxia**	0.7681	0.8688	0.7646	0.7198	0.6956	0.6033	0.6632	0.8289	0.6933	0.7264	0.7224	0.6852	0.7319	0.7286	10
**Qinghai**	0.3378	0.4099	0.4825	0.4484	0.4187	0.4384	0.5419	0.5561	0.5763	0.7112	0.8404	0.7432	0.8369	0.5648	11
**Xinjiang**	0.7873	0.7664	0.7578	0.7912	0.8599	0.9708	0.8545	0.8623	0.8953	1.0000	1.0000	1.0000	1.0000	0.8881	8
**Inner Mongolia**	0.7414	0.7453	0.7701	0.8281	0.9195	1.0000	1.0000	1.0000	0.9498	0.9340	1.0000	0.9443	1.0000	0.9102	7

In terms of average technical efficiency, provinces such as Shaanxi (1), Chongqing (0.9946), Sichuan (0.9928), and Gansu (0.9772) exhibited relatively high average efficiency values. This indicates that these provinces effectively convert existing resources and technology into outputs, demonstrating high levels of resource utilization and management. It suggests that higher education in these provinces is near the production frontier, with output levels approaching the optimal values for the current inputs, thus leaving limited room for further efficiency improvements. Notably, Shaanxi, with an average technical efficiency score of 1, showed no efficiency loss in its higher education sector, with no redundancy in inputs or outputs, indicating strong DEA efficiency. In contrast, provinces such as Tibet (0.3828), Qinghai (0.5648), Ningxia (0.7286), and Guizhou (0.8416) had lower scores, implying that higher education in these regions have not effectively transformed inputs into outputs under the current resource and technological conditions. These regions have yet to reach the production frontier, revealing issues of resource wastage and insufficient management efficiency that urgently require improvement.

(2) Decomposition analysis of technical efficiency.

To further analyze the specific components of input-output efficiency in higher education within western China and to objectively assess whether the allocation of inputs and outputs is near an optimal configuration, the study applies the decomposition formula “TE = PTE × SE” to examine the average values of technical efficiency, pure technical efficiency, and scale efficiency for western China from 2010 to 2022. In addition, the study compares the results from the first and third phases. The decomposition results are presented in Table 6, S4 Table and S5 Table.

**Table 6:** Decomposition of Technical Efficiency in First and Third Stages.

Province	Technical Efficiency Score and Rank		Pure Technical Efficiency Score and Rank		Scale Efficiency Score and Rank	
	First Stage	Third Stage	First Stage	Third Stage	First Stage	Third Stage
Chongqing	1.000 (1)	0.9946 (2)	1.000 (1)	0.9992 (3)	1.000 (1)	0.9954 (2)
Sichuan	0.9832 (4)	0.9928 (3)	1.000 (1)	1.000 (1)	0.9832 (9)	0.9928 (3)
Yunnan	0.9867 (3)	0.9760 (5)	0.9955 (5)	0.9865 (6)	0.9911 (7)	0.9889 (4)
Guizhou	0.8550 (12)	0.8416 (9)	0.8568 (12)	0.8940 (12)	0.9978 (5)	0.9417 (7)
Guangxi	0.9528 (8)	0.9556 (6)	0.9536 (10)	0.9677 (11)	0.9991 (3)	0.9873 (6)
Tibet	0.9005 (10)	0.3828 (12)	0.9991 (4)	0.9709 (10)	0.9014 (12)	0.3943 (12)
Shaanxi	1.000(1)	1.000 (1)	1.000 (1)	1.000 (1)	1.000 (1)	1.000 (1)
Gansu	0.9772 (6)	0.9772 (4)	0.9785 (7)	0.9894 (4)	0.9985 (4)	0.9874 (5)
Ningxia	0.8858 (11)	0.7286 (10)	0.9238 (11)	0.9869 (5)	0.9569 (10)	0.7380 (10)
Qinghai	0.9256 (9)	0.5648 (11)	0.9768 (8)	0.9722 (8)	0.9463 (11)	0.5778 (11)
Xinjiang	0.9817 (5)	0.8881 (8)	0.9910 (6)	0.9751 (7)	0.9905 (8)	0.9091 (9)
Inner Mongolia	0.9654 (7)	0.9102 (7)	0.9699 (9)	0.9716 (9)	0.9954 (6)	0.9351 (8)
Mean	0.9512	0.8510	0.9704	0.9761	0.9800	0.8707

Overall, the pure technical efficiency (average of 0.9761) of higher education in western China during the third stage demonstrated a favorable performance. However, both technical efficiency (average of 0.8510) and scale efficiency (average of 0.8707) remained relatively low, indicating issues of suboptimal resource allocation and inadequate management within higher education, with scale inefficiency further constraining potential improvements in technical efficiency. Following adjustments to the input variables, DMUs with initially lower efficiency scores saw further declines, suggesting that the disparity in input-output efficiency across higher education in western China widened after controlling for external environmental effects.

Analyzing the adjusted technical efficiency values, Tibet, Qinghai, and Ningxia notably fell below the mean, reflecting substantial inefficiencies related to management or scale limitations in their higher education sectors. The adjusted pure technical efficiency indicated that most provinces approached optimal technical management within their existing scale, demonstrating high technical effectiveness. Notably, Shaanxi and Sichuan had pure technical efficiency scores of 1, signifying that higher education in these provinces has achieved optimal levels in technical management and resource utilization. In contrast, Guizhou exhibited the lowest pure technical efficiency score, while Guangxi, Tibet, Inner Mongolia, Qinghai, and Xinjiang also scored below the average, indicating that these provinces have not fully leveraged resources under their current scale, resulting in technical and managerial inefficiencies. The adjusted scale efficiency revealed that scale inefficiency persisted across most provinces. Shaanxi, Chongqing, and Sichuan had scale efficiency values equal to or near 1, suggesting that higher education in these provinces has largely attained optimal scale, avoiding significant resource wastage or scale misalignment. Conversely, Tibet, Qinghai, and Ningxia reported the lowest scale efficiency scores at 0.3943, 0.5778, and 0.7380, respectively, indicating severe scale inefficiency. Further analysis of the third-stage DEA results revealed that these provinces operated under increasing returns to scale, implying that enhancing input-output efficiency in higher education could be achieved through scale expansion and optimized resource allocation.

(3) Comparative analysis of efficiency between the first and third stages.

Table 6, in conjunction with S1-S3 Figs, presents a comparative analysis of the initial DEA efficiency values from the first stage and the adjusted efficiency values from the third stage. The average technical efficiency in the first stage was 0.9512, but following the three-stage DEA adjustment, this average decreased to 0.8510. The decline in technical efficiency for most provinces after adjustment indicates that initial measurements overestimated the actual technical efficiency. Tibet exhibited the largest reduction, with technical efficiency dropping from 0.9005 to 0.3828, followed by Qinghai and Ningxia, suggesting that the technical efficiency in these three provinces heavily depends on favorable external conditions. After excluding these factors, the actual input-output efficiency of higher education in these western provinces proves lower than initially estimated.

The average pure technical efficiency in the first stage was 0.9704, which slightly increased to 0.9761 after the third-stage adjustment. This suggests that, after controlling for external environmental factors, the pure technical efficiency in higher education across most provinces remains relatively stable, indicating that the technical management level is not significantly influenced by external conditions. Notably, Shaanxi and Sichuan maintained a pure technical efficiency of 1, further demonstrating that their optimal level of technical management was unaffected by external environmental factors.

The initial average scale efficiency was 0.98, nearing optimal levels. Nonetheless, following the third-stage adjustment, the average scale efficiency declined to 0.8707, highlighting the role that favorable external conditions initially played in supporting scale efficiency. After adjustment, scale efficiency declined to varying degrees in most provinces (with the exception of Sichuan and Shaanxi), particularly in Tibet, Qinghai, and Ningxia, where scale efficiency fell from 0.9014, 0.9463, and 0.9569 to 0.3943, 0.5778, and 0.7380, respectively. This indicates that the scale of higher education in these regions is far from optimal, with pronounced scale inefficiency. Factors such as sparse population, weak economic foundations, and underdeveloped infrastructure contribute to a mismatch between resource inputs and outputs. Once favorable external conditions were excluded, the scale inefficiency issues in regions like Tibet, Qinghai, and Ningxia became more pronounced, revealing an urgent need for optimized adjustments in scale.

(4) Overall trend analysis.

**Fig 1:**
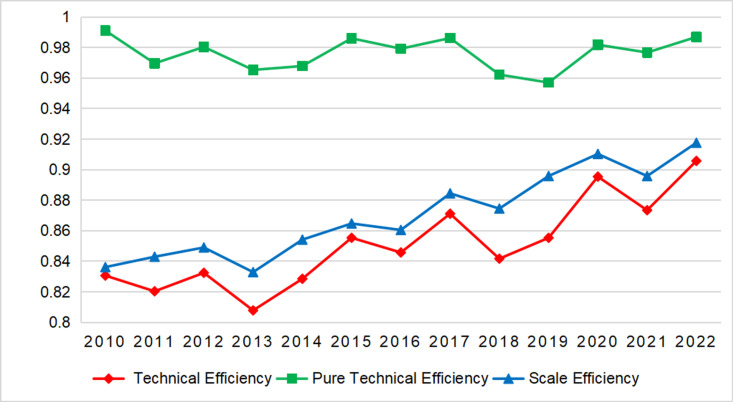
Trends in TE, PTE, and SE in Western China (2010-2022).

The trends in technical efficiency, pure technical efficiency, and scale efficiency of higher education in western China from 2010 to 2022 are illustrated in Fig 1.

Overall, from 2010 to 2022, the technical management capabilities of higher education in western China have shown a steady improvement, although scale inefficiency remains a key constraint on technical efficiency. Technical efficiency has exhibited a fluctuating upward trend since 2010, reaching its peak of 0.9057 in 2022. Pure technical efficiency has remained consistently high throughout the study period, with an average of 0.9761, and notably sustained levels above the average during 2015–2017 and 2020–2022, reflecting relatively mature management and technical operations within the region’s higher education sector. In contrast, scale efficiency has been comparatively lower over these 13 years, with an average of 0.8707. Although scale efficiency has also shown a fluctuating upward trend, its fluctuations are less pronounced than those of technical efficiency, with a noticeable improvement beginning in 2018, indicating a gradual resolution of scale inefficiency issues in recent years. However, despite the upward trend, from a time-series perspective, scale inefficiency remains the primary bottleneck restricting overall technical efficiency gains. This suggests that in certain provinces within western China, economies of scale in higher education have not yet been fully realized, underscoring an urgent need to optimize scale in order to enhance resource utilization efficiency and thereby promote improvements in overall technical efficiency.

### 5.2 Empirical results of global Malmquist productivity index

Building on the efficiency analysis of DEA using the global benchmark technology presented earlier, this section employs the GMI to decompose changes in TFP, thereby assessing temporal variations in higher education productivity in western China and capturing the causes for these changes. Specifically, the GMI is expressed as GMI = TC × EC = TC × (PEC × SEC). The annual TFP and its decomposed indices for western China are presented in Table 7 and S4 Fig.

**Table 7:** Annual GMI and Decomposition Indices for Western China.

Year	EC	TC	PEC	SEC	GMI
**2010-2011**	0.9910	1.0038	1.0000	0.9910	0.9948
**2011-2012**	0.9825	1.0428	1.0000	0.9825	1.0246
**2012-2013**	0.9938	0.9716	0.9960	0.9978	0.9656
**2013-2014**	1.0119	1.0136	0.9965	1.0155	1.0257
**2014-2015**	0.9885	1.0430	1.0061	0.9826	1.0311
**2015-2016**	0.9997	0.9998	0.9976	1.0022	0.9995
**2016-2017**	1.0619	0.9799	1.0003	1.0616	1.0405
**2017-2018**	0.9988	0.9625	1.0036	0.9952	0.9613
**2018-2019**	1.0580	0.9699	1.0000	1.0580	1.0261
**2019-2020**	0.9965	1.0480	1.0000	0.9965	1.0443
**2020-2021**	0.9858	0.9926	0.9985	0.9872	0.9785
**2021-2022**	1.0087	1.0301	1.0015	1.0072	1.0390
**Mean**	1.0061	1.0044	1.0000	1.0061	1.0105

As shown in Table 7, the mean annual GMI for higher education in western China was 1.0105, indicating an overall upward trend in input-output performance for the period 2010–2022, with an average annual growth of 1.05%. During the period, GMI experienced three notable fluctuations, with the most significant occurring between 2016 and 2020. The lowest GMI value of 0.9613 was observed from 2017 to 2018, reflecting the sharpest productivity decline in this interval. The other two low values appeared in 2012–2013 (0.9656) and 2020–2021 (0.9785). Conversely, GMI reached a peak of 1.0443 in 2019–2020. This peak indicates that the higher education system exhibited its highest level of productivity during this period, with a marked enhancement in educational output capacity.

The technical efficiency change (EC) measures variations in the efficiency of resource utilization within HEIs at a given technological level. An EC value exceeding 1 suggests an enhancement in relative technical efficiency, while an EC value less than 1 demonstrates a deterioration in relative technical efficiency. Over the study period, the average EC was 1.0061, suggesting a general upward trend in technical efficiency change, with an annual growth of approximately 0.61%. The years 2016–2017 (1.0619) and 2018–2019 (1.0580) marked the most significant improvements in technical efficiency, with annual increases of 6.19% and 5.80%, respectively, reflecting notable advances in resource utilization and management during these periods. Except for the years 2013–2014, 2016–2017, 2018–2019, and 2021–2022, EC values in other years were below 1, indicating a decline in relative technical efficiency. As a whole, the mean of EC surpasses that of TC, suggesting that EC is the principal driver of TFP improvement. This finding is further substantiated by recent studies on productivity shifts within both Europe’s and China’s higher education system, which collectively conclude that efficiency change, rather than technological improvement, represents the dominant force for productivity growth in this sector [15,16,87], in contrast to evidence derived from British and Australian experiences, which suggests a predominant role for technological change in driving overall TFP growth in HEIs [63,164,165].

Technological change (TC) represents the shift in the production frontier, representing either advancement or regression in the technological frontier, primarily driven by technological innovation. A TC value above 1 signifies technological progress, while a TC value below 1 denotes technological regression. Over the study period, the average TC value was 1.0044, suggesting an annual increase in technological level of approximately 0.44%. Notably, during 2019–2020 (1.0480), 2014–2015 (1.0430), and 2011–2012 (1.0428), the TC index was relatively high, indicating significant technological progress in these years. Conversely, in years such as 2012–2013 and 2017–2018, the TC index fell below 1, indicating some degree of technological regression. During 2013–2014 and 2021–2022, both EC and TC exceeded 1, with TC surpassing EC, resulting in a GMI greater than 1. This demonstrates that technological progress contributed more to TFP during these periods than did improvements in technical efficiency, emerging as the main driver of productivity growth. In 2011–2012, 2014–2015, and 2019–2020, although EC fell below 1, reflecting a decline in technical efficiency, TC remained above 1, maintaining GMI at a level above 1. This demonstrates that technological progress not only offset the decline in technical efficiency but also acted as a critical factor driving TFP growth in these years, underscoring the essential role of technological advancement in enhancing productivity within higher education.

Pure technical efficiency change (PEC) measures shifts in the efficiency of resource allocation and management within HEIs under a given level of technology. It reflects whether, within existing technological constraints, HEIs have improved output efficiency through more effective resource allocation or management optimization. Over the study period, the mean PEC value was 1, indicating that, generally, resource allocation efficiency and management systems in western HEIs remained stable, with no significant changes. Specifically, in 2014–2015, 2017–2018, and 2021–2022, PEC values exceeded 1, suggesting that improvements in management practices or resource allocation occurred in these years within western HEIs. Conversely, in 2012–2013, 2013–2014, 2015–2016, and 2020–2021, PEC values were below 1, indicating a decline in resource allocation or management efficiency during these years, revealing issues of mismanagement or suboptimal resource allocation.

Scale Efficiency Change (SEC) measures whether a production unit has reached the optimal production scale, reflecting the influence of economies of scale on the production frontier. An SEC value exceeding 1 indicates an enhancement in scale efficiency, suggesting that the DMU operates closer to the optimal production scale; an SEC value below 1 denotes a decline in scale efficiency, signaling that the DMU’s production scale deviates from the optimal level. The mean SEC value of 1.0061 suggests that, on average, the scale efficiency of HEIs in western China increased by approximately 0.61% per year over the study period. This implies that SEC was a critical factor in driving TFP growth. Specifically, in 2013–2014, 2015–2016, 2016–2017, 2018–2019, and 2021–2022, SEC values exceeded 1, indicating that the production scale of western HEIs approached an optimal state during these years. Particularly in 2016–2017 (1.0616) and 2018–2019 (1.0580), SEC values were at their highest, reflecting the most significant improvements in scale efficiency. However, in other years, SEC values fell below 1, indicating a decline in scale efficiency and suggesting that HEI scales in western China failed to reach optimal configurations. In western China, multiple factors—such as relatively low economic development levels, low population density, fewer students of school age, insufficient public resources, and limited financial support—collectively constrain the expansion of university scale, preventing the achievement of economies of scale and thereby impacting scale efficiency.

In summary, SEC has been pivotal in improving TFP. From 2010 to 2022, TFP and SEC in western HEIs have shown a modest upward trend. However, in certain years, the SEC index declined, particularly after 2019, with a relatively smaller increase in scale efficiency, indicating that western HEIs still need to optimize resource allocation and adjust their scale structures to achieve optimal production scales. Simultaneously, the contribution of TC to TFP should not be overlooked. Although TC’s contribution to TFP has been slightly lower than that of EC overall, in some years, technological progress surpassed improvements in technical efficiency, becoming the primary driver of TFP growth. Therefore, in promoting TFP growth in western HEIs, it is essential to not only focus on the enhancement of scale efficiency within technical efficiency but also to emphasize the long-term role of technological progress to ensure the sustainable development of higher education in western China.

Similarly, the GMI is further employed to evaluate the dynamic efficiency of higher education input-output across provinces in western China, yielding the TFP index and its decomposed indices for each province, as presented in Table 8 and S5 Fig.

**Table 8:** GMI and Decomposition Indices for Each Province in Western China.

Province	EC	TC	PEC	SEC	GMI
**Chongqing**	1.0000	1.0000	1.0000	1.0000	1.0000
**Sichuan**	1.0000	1.0000	1.0000	1.0000	1.0000
**Yunnan**	1.0000	1.0000	1.0000	1.0000	1.0000
**Guizhou**	1.0000	0.9862	1.0000	1.0000	0.9862
**Guangxi**	1.0000	1.0000	1.0000	1.0000	1.0000
**Tibet**	0.9940	1.0279	1.0000	0.9940	1.0217
**Shaanxi**	1.0000	1.0000	1.0000	1.0000	1.0000
**Gansu**	1.0000	1.0015	1.0000	1.0000	1.0015
**Ningxia**	0.9980	0.9980	1.0000	0.9980	0.9960
**Qinghai**	1.0705	1.0075	1.0000	1.0705	1.0785
**Xinjiang**	1.0132	1.0069	1.0000	1.0132	1.0201
**Inner Mongolia**	1.0000	1.0252	1.0000	1.0000	1.0252
**Mean**	1.0061	1.0044	1.0000	1.0061	1.0105

The annual average GMI values for Qinghai (1.0785), Inner Mongolia (1.0252), Tibet (1.0217), Xinjiang (1.0201), and Gansu (1.0015) all exceeded 1, suggesting a significant improvement in the productivity of higher education in these provinces over the course of the study period. Notably, Qinghai exhibited the most substantial increase, achieving an average annual growth rate of 7.85%. Conversely, the GMI values for Guizhou (0.9862) and Ningxia (0.9960) fell below 1, indicating a decline in higher education productivity in these provinces, with Guizhou experiencing an average annual decrease of 1.38% and Ningxia 0.4%. The GMI values for other provinces remained at 1, indicating stable higher education productivity with no significant changes observed throughout the study period.

Regarding EC, most provinces exhibited an EC value of 1, indicating stable technical efficiency throughout the study period with no significant improvements or declines. Consequently, the contribution of technical efficiency to TFP in these provinces is relatively limited. This suggests that in much of the western region, HEIs have not experienced notable improvements or deteriorations in resource utilization efficiency, management, or operational capability at the current technological level. Furthermore, changes in resource supply, educational demand, or policy adjustments have not exerted sufficient influence to alter technical efficiency within the higher education sector. Notably, Qinghai (1.0705) and Xinjiang (1.0132) demonstrated improvements in technical efficiency, with Qinghai showing the most significant gains, suggesting progress in resource utilization efficiency or positive impacts from relevant policy adjustments. Conversely, the EC values for Ningxia (0.9980) and Tibet (0.9940) were slightly below 1, suggesting a decline in resource allocation and management efficiency in these areas, highlighting a need for further optimization of management practices.

Concerning TC, Tibet (1.0279), Inner Mongolia (1.0252), and Gansu (1.0015) demonstrated noticeable technological progress, with Tibet and Inner Mongolia particularly distinguished in technological innovation and advancements in production techniques. In contrast, Guizhou (0.9862) and Ningxia (0.9980) had TC values below 1, indicating technological regression, suggesting the need to strengthen research investment and innovation capacity to address their lag in technological progress.

All provinces exhibited a PEC value of 1, indicating stable resource allocation efficiency under existing technological conditions during the study period, with no significant changes observed. This finding suggests that while western HEIs have made no substantial advances in resource allocation at the current technology level, there has also been no decline in resource utilization efficiency.

With respect to SEC, Qinghai (1.0705) and Xinjiang (1.0132) showed the most significant scale efficiency improvements, suggesting that the scale of higher education in these provinces is accelerating toward optimization. Qinghai, in particular, achieved an average annual increase in scale efficiency of 7.05%. Conversely, the SEC values for Tibet (0.9940) and Ningxia (0.9980) were below 1, indicating that higher education in these provinces had not yet reached optimal scale efficiency, necessitating further optimization of resource allocation or adjustments in institutional scale.

In summary, Qinghai, Inner Mongolia, Tibet, Xinjiang and Gansu exhibited significant TFP improvement during the study period, with Qinghai standing out in particular. However, Guizhou and Ningxia showed a downward trend in TFP, suggesting the need for enhanced management and technological innovation. In Tibet, Inner Mongolia, and Gansu, technological progress is the primary driver of TFP growth, whereas in Qinghai and Xinjiang, TFP growth is primarily attributable to improvements in scale efficiency. The decline in TFP in Guizhou is primarily attributed to technological regression, while Ningxia’s decline is affected by both technological regression and decreased scale efficiency. Additionally, there remains room for further optimization in scale efficiency in Tibet.

### 5.3 Empirical results of Tobit regression

#### 5.3.1 Analysis of Tobit regression results.

In the Tobit model, the dependent variable is the adjusted technical efficiency measured in the third-stage DEA, while the independent variables include the proportion of regional fiscal revenue to gross regional product (RFR/GRP), the proportion of full-time faculty with senior and associate senior academic titles (SAS/FTF), the proportion of employees with college education or above (PECE), per-student education expenditure (PSEE), and the proportion of higher education operating expenses to total education expenditure (HEOE/TEE). Using Stata 18 software, a Tobit regression analysis was conducted to explore the factors influencing higher education input-output efficiency in western China, with the results displayed in Table 9.

**Table 9:** Tobit Regression Results.

Dimension	Variables	Coefficients	Std. Errors	t-value	p-value	95% CI
Socio-economic Environment	RFR/GRP	−0.0028	0.0067	−0.42	0.675	[-0.0159514, 0.0103585]
Human Capital	SAS/FTF	−0.0162	0.0044	−3.65	0.000^***^	[-0.0249552, -0.0074328]
	PECE	0.0179	0.0040	4.48	0.000^***^	[0.0099784, 0.0257519]
Educational Funding	PSEE	−7.53E-06	2.00E-06	−3.77	0.000^***^	[-0.0000115, -3.59e-06]
	HEOE/TEE	0.0126	0.0026	4.83	0.000^***^	[0.0074507, 0.0177687]
	Constant	0.9269	0.2535	3.66	0.000^***^	[0.4260967, 1.427753]

Note: *, **, *** indicate significance at the 10%, 5%, and 1% statistical levels, respectively.

According to the regression results in Table 9, the regression coefficients, standard errors, t-values, p-values, and 95% confidence intervals for the key explanatory variables clearly illustrate the impact of each variable on the dependent variable and their respective significance levels.

Firstly, the regression coefficient of RFR/GRP is −0.0028, indicating a negative relationship between the proportion of regional fiscal revenue to GRP and the technical efficiency of higher education input-output. However, with a p-value of 0.675, this variable does not reach a level of statistical significance, suggesting that in this model, the influence of RFR/GRP on the technical efficiency of higher education input-output is not statistically significant, and its explanatory power within the study sample is relatively weak.

Secondly, the regression coefficient for SAS/FTF is −0.0162, with a significance level of p < 0.01, indicating that an increase in the proportion of full-time faculty with senior academic titles (full and associate professorships) has a significantly negative impact on the technical efficiency of higher education input-output. Although senior and associate senior faculty members typically possess strong research and teaching capabilities, in the resource-constrained western China, a higher proportion of senior faculty may lead to imbalanced resource allocation. This finding contrasts with the conclusions of [97,103,119], which assert that the proportion of full-time faculty holding senior academic titles exerts a significantly positive impact on the technical efficiency of HEIs. Three main reasons underpin our finding. Primarily, although the Chinese government has introduced the “Western Development” strategy, with central and local governments actively promoting the expansion of tertiary education, encouraging talent return, and strengthening local high-level talent cultivation, HEIs in western China have not effectively allocated these human resources in the recruitment and cultivation of senior faculty. Resources tend to concentrate in economically more developed cities, key institutions, and certain fields, leading to further centralization of educational resources within the region, ultimately failing to benefit all HEIs and diminishing overall educational resource utilization efficiency. Additionally, the relatively higher salaries and benefits associated with senior faculty positions further exacerbate resource allocation imbalances. While a higher proportion of senior faculty can enhance certain teaching and research outcomes, if not managed effectively—particularly in the resource- and funding-limited western China—an excessively high proportion of senior faculty may generate increased management costs, thereby hindering improvements in higher education input-output efficiency. Moreover, senior faculty members often undertake managerial roles or academic leadership responsibilities in addition to their teaching and research duties. The excessive involvement in administrative tasks can detract from their direct contributions to teaching and research, resulting in a decline in technical efficiency. The negative correlation between the proportion of senior faculty and technical efficiency does not imply inefficiency on the part of senior faculty themselves but rather reflects issues stemming from traditional management structures and suboptimal task allocation.

Thirdly, the regression coefficient for PECE is 0.0179, with a significance level of p < 0.01, indicating that the proportion of employees with college education or above has a significant positive impact on the improvement of technical efficiency in higher education in western China. Attributable to factors such as geographic location and economic foundations, western China has historically faced relatively unequal distribution of educational resources, with underdeveloped education levels and limited talent development serving as bottlenecks to economic and social progress. However, with the increased governmental investment in education in western China, especially in higher education, the quality of higher education has enhanced significantly, greatly facilitating human capital accumulation. From the perspective of human capital theory, education is a critical investment in enhancing the productivity and innovation capacity of the workforce. By increasing investment in higher education, the western region not only develops a labor force with advanced professional skills and innovation abilities but also enhances the adaptability of workers to new technologies and industries—essential factors for driving economic transformation and improving overall productivity. Workers with higher education attainment demonstrate greater efficiency in economic activities, fostering modernization within organizations and industries and strengthening regional competitiveness. Therefore, the significant positive coefficient of PECE reflects that, with the growing accessibility of higher education in western China, the proportion of well-educated labor has risen, accelerating human capital accumulation. This process directly contributes to the improvement of technical efficiency in higher education in the region. In other words, the enhancement of overall regional human resource levels has played a crucial role in advancing higher education development.

Fourthly, the regression coefficient for PSEE is −7.53E-06, with a high level of significance (p < 0.01), indicating a negative relationship between per-student education expenditure and higher education input-output technical efficiency. This result suggests a phenomenon of diminishing marginal returns in the increase of per-student educational funding in western China. Although the government, through the “Double First-Class” initiative and other policy-based funding support, aims to enhance the teaching quality and research level of HEIs in western China, the effective utilization of these funds may face challenges. In many cases, the substantial input in per-student education expenditure has not directly translated into tangible educational outcomes but has been diverted to infrastructure projects or unnecessary expansions, resulting in low efficiency in fund utilization. Simultaneously, given the underdeveloped management systems and oversight mechanisms for fund allocation, the increased per-student education expenditure may lead to resource wastage or overexpansion of certain projects that exceed the actual educational needs of western China, thus inflating management costs and suppressing overall technical efficiency. This phenomenon also highlights that, while financial support for education in western China continues to increase, significant room for improvement remains in the rational allocation and utilization of these funds to maximize input-output efficiency. For HEIs in western China, which benefit from favorable national policies and financial support, the effective application of funds to improve teaching quality, facilitate research outcomes, and cultivate talent should be a primary focus of future policy and management efforts. This finding is reinforced by a recent study, which indicates that current governmental financial support for universities may be sufficient, and that indiscriminate increases in financial investment are likely to yield limited improvements in performance [166].

Fifthly, the regression coefficient for HEOE/TEE is 0.0126, with a significance level of p < 0.01, indicating that the proportion of higher education operating expenses to total education expenditure has a significant positive impact on improving the technical efficiency of higher education in western China. When the share of higher education operating expenses within total educational spending increases, resources are allocated and utilized more effectively, thereby improving the overall operational efficiency of the educational system. Higher education operating expenses are typically allocated to infrastructure development, equipment procurement, and faculty training, all of which lay a foundation for improved educational quality and long-term benefits. A higher proportion of operating expenses ensures that educational resources are efficiently distributed and utilized, thereby fostering efficiency improvements.

Overall, SAS/FTF, PECE, PSEE, and HEOE/TEE are statistically significant at the 1% level, reflecting the substantial impact of human capital structure and educational funding allocation on the input-output efficiency of higher education in western China. The positive significance of HEOE/TEE and PECE underscores the importance of higher education operating expenses and external human capital, while the negative significance of SAS/FTF and PSEE suggests potential issues of diminishing marginal returns in faculty utility and resource misallocation.

#### 5.3.2 Robustness test.

To verify the robustness of the regression results, this study employs Tobit regression with robust standard errors and Probit regression as comparative analysis, with the results shown in Table 10.

**Table 10:** Regression results: Tobit model with robust SE and probit Model.

Variables	Tobit with Robust Standard Errors				Probit			
	Coefficients	Robust Std. Errors	t-value	p-value	Coefficients	Std. Errors	t-value	p-value
RFR/GRP	−0.0028	0.0068	−0.41	0.681	−0.0425	0.0414	−1.03	0.304
SAS/FTF	−0.0162	0.0057	−2.84	0.005^***^	−0.0733	0.0270	−2.71	0.007^***^
PECE	0.0179	0.0040	4.49	0.000^***^	0.0717	0.0272	2.63	0.008^***^
PSEE	−7.53E-06	1.89E-06	−3.98	0.000^***^	−5.96E-05	1.84E-05	−3.23	0.001^***^
HEOE/TEE	0.0126	0.0025	5.12	0.000^***^	0.0364	0.0173	2.11	0.035^**^
Constant	0.9269	0.3418	2.71	0.007^***^	2.7725	1.5058	1.84	0.066

Note: *, **, *** indicate significance at the 10%, 5%, and 1% statistical levels, respectively.

Comparing Tables 10 and 9 reveals a high degree of consistency between the Tobit regression with robust standard errors and the standard Tobit regression results. The coefficient direction, magnitude, and significance levels of the main explanatory variables (SAS/FTF, PECE, PSEE, HEOE/TEE) remain largely unchanged. The Probit regression results are also broadly consistent with those of the Tobit regression. The significance level of the variable HEOE/TEE differs slightly between the Probit and Tobit models, showing higher significance in the Tobit model (p < 0.01) compared to the Probit model (p < 0.05). Nonetheless, the effect direction of HEOE/TEE is consistent across both models and remains significant, indicating a robust positive effect of HEOE/TEE on the dependent variable in both models. Additionally, there is a minor difference in the significance of the constant term between the Probit and Tobit models: it is insignificant in the Probit model, while the explanatory power of the overall model and the impact of the variables remain stable. Through the comparison with the Probit model and the introduction of robust standard errors in the Tobit model, the study achieves highly consistent regression results, particularly regarding the significance and direction of the main explanatory variables. Therefore, it can be concluded that the regression results in this study exhibit robustness and reliability.

## 6. Conclusion and policy implications

This study employs a three-stage DEA and the global Malmquist index to evaluate and analyze the input-output efficiency and productivity changes in higher education across western China from both static and dynamic viewpoints. A Tobit model is further used to identify the factors influencing technical efficiency. Drawing upon the empirical findings, the following conclusions can be inferred.

Firstly, environmental variables and random errors significantly impact the input-output efficiency of higher education in western China, resulting in an overestimation of overall efficiency. After controlling for environmental factors, the mean values of technical efficiency and scale efficiency for higher education in western China decrease substantially, while the mean value of pure technical efficiency shows a slight increase. The adjusted mean scores for technical efficiency, pure technical efficiency, and scale efficiency are 0.8510, 0.9761, and 0.8707, respectively.

Secondly, there are significant interprovincial disparities in higher education efficiency across western China. During the sample period, only Shaanxi achieved DEA effectiveness in technical efficiency, followed closely by Chongqing, whereas Tibet, Qinghai, and Ningxia had relatively low scores, with a considerable gap between the highest- and lowest-scoring provinces. Most western provinces demonstrate relatively high levels of pure technical efficiency; however, deficiencies in scale efficiency have lowered overall technical efficiency, particularly in provinces such as Tibet, Qinghai, and Ningxia, where scale inefficiency is especially pronounced.

Thirdly, the TFP in western China has achieved a modest annual growth rate of 1.05%. The increase in TFP has been driven by both improvements in technical efficiency and technological progress, with the greater contribution coming from enhancements in technical efficiency, largely caused by improvements in scale efficiency. At the provincial level, Qinghai, Xinjiang, Inner Mongolia, Tibet, and Gansu have seen significant increases in TFP, with Qinghai performing particularly well. In contrast, Guizhou and Ningxia have experienced a downward trend in TFP. Technological progress serves as the principal factor contributing to TFP growth in Tibet, Inner Mongolia, and Gansu, whereas the growth in TFP in Qinghai and Xinjiang mainly stems from improvements in scale efficiency. The decline in TFP in Guizhou is primarily due to technological regression, while Ningxia’s decrease is influenced by both technological regression and a reduction in scale efficiency.

Finally, both the internal and external structures of human capital and the allocation of educational funding significantly impact the technical efficiency of higher education in western China. Specifically, the proportion of full-time faculty with senior and associate senior academic titles and per-student education expenditure are significantly negatively correlated with technical efficiency, whereas the proportion of operating expenses to total higher education expenditure and the proportion of employees with college education or above are significantly positively correlated with technical efficiency. This indicates that the structural optimization of human resources and financial allocation is essential for enhancing higher education efficiency.

The study recommends a range of policy adjustments to improve the input-output efficiency of higher education in western China. (1) Provide targeted support for regionally differentiated higher education development. Significant interprovincial disparities in higher education efficiency across western China require tailored support from both central and local governments. Provinces with insufficient scale efficiency need optimized resource allocation, institutional size adjustments, and increased resource sharing. For Qinghai, where technical efficiency has notably improved, efforts should focus on scaling adjustments while promoting technological progress and innovation. Tibet, which has the lowest scores in both technical efficiency and the technical efficiency change index, requires optimized resource utilization, expanded educational scale in line with regional needs, stronger external support, and better employment guidance. For Ningxia and Guizhou, where total factor productivity has declined, addressing technological regression should take precedence, with interventions spanning research investment, technological innovation, management system improvement, and external resource mobilization. (2) Enhance modernized management to drive scale efficiency and technological advancement. The sustainable development of higher education in western China depends on the synergistic improvement of scale efficiency and technological advancement. HEIs should strengthen regional resource integration, facilitate shared use of research platforms, laboratories, and teaching facilities to avoid redundancy, and align institutional scales with regional socio-economic demand. Simultaneously, further efficiency gains can be realized via technological innovation. Through the convergence of information technology and intelligent management systems, HEIs can establish a scientifically grounded resource input framework that enables accurate forecasting, dynamic adjustment, and precise allocation of resource volume and composition. This approach tailors inputs to institutional types, disciplinary characteristics, and regional variations, thereby refining the level of resource allocation efficiency and minimizing redundancies. (3) Optimize funding structure to enhance the efficiency of fund utilization. Continued financial support for higher education in western China is essential, but optimizing the allocation structure is key to improving fund utilization. Increasing the proportion of operational expenses helps support the efficient functioning of teaching and research. Compared to research-specific funds, operational funding provides more immediate and sustainable benefits. Setting reasonable standards of per-student education expenditure helps align funding with actual needs and reduce waste. Establishing a performance evaluation mechanism ensures funds are allocated to critical areas of teaching and research. Furthermore, western HEIs should establish a diversified funding input mechanism and expand non-fiscal funding sources through engaging in research services and university-industry collaborations, thereby enhancing the overall fund efficiency. (4) Dual pathways to unlock human capital potential: optimizing faculty and regional human resources. Rational faculty composition is crucial for enhancing higher education efficiency. The negative correlation between the proportion of senior faculty and technical efficiency suggests that rigid rank structures and suboptimal role distribution can lead to inefficiencies. A “dynamic role allocation” mechanism should be implemented to flexibly adjust the responsibilities of senior faculty in teaching, research, and administration according to project and disciplinary needs. This fully leverages faculty potential and prevents mismatches and efficiency losses from fixed roles. At the same time, it is essential to maintain a balanced ratio of faculty ranks and foster collaboration, ensuring faculty at all levels contribute effectively within their expertise, thus preventing imbalances in human resource structures. In addition to internal university optimization, efforts must also focus on enhancing overall human capital at the regional level. Strategies should focus on actively recruiting talent with advanced degrees and specialized skills, implementing more attractive compensation and benefits, strengthening academic support and career development pathways, and providing a supportive work environment. These measures will increase the appeal of western HEIs to high-level talent, reduce attrition, and reinforce local talent development, thereby supporting the sustained advancement of higher education.

## Supporting information

S1 Fig A comparison between the TE values from the first stage and the third stage. (TIF)

S2 Fig A comparison between the PTE values from the first stage and the third stage. (TIF)

S3 Fig A comparison between the SE values from the first stage and the third stage. (TIF)

S4 Fig Line chart of annual trends of GMI, EC, TC, PEC, and SEC. (TIF)

S5 Fig The indices of GMI, EC, TC, PEC and SEC of 12 provinces in western China. (TIF)

S1 Table Summary of recent relevant literature on input-output indicators in higher education. (DOCX)

S2 Table Initial pure technical efficiency of higher education in western China (2010–2022). (DOCX)

S3 Table Initial scale efficiency of higher education in western China (2010–2022). (DOCX)

S4 Table Adjusted pure technical efficiency of higher education in western China (2010–2022). (DOCX)

S5 Table Adjusted scale efficiency of higher education in western China (2010–2022). (DOCX)

S6 Table Original input-output data. (XLSX)

S7 Table First-stage TE values with 156 DMUs. (XLSX)

S8 Table First-stage TE values with Global DEA. (XLSX)

S9 Table First-stage TE values with traditional DEA. (XLSX)
