# Supplementary figures and images for: Input-output efficiency, productivity dynamics, and determinants in western China’s higher education: A three-stage DEA, global Malmquist index, and Tobit model approach
Source: PLoS One. 2025 Jun 11;20(6):e0325901. doi: 10.1371/journal.pone.0325901 (PMC12157086; doi:10.1371/journal.pone.0325901)

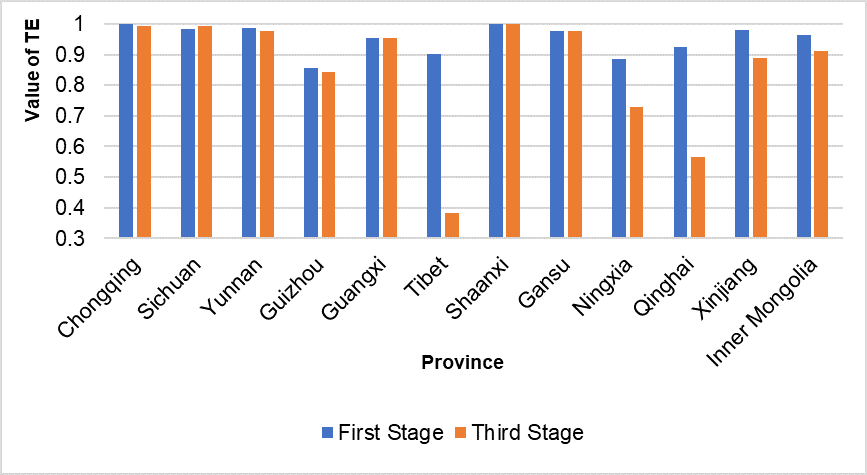

Supplement: S1 Fig — (TIF) [file pone.0325901.s001.tif]

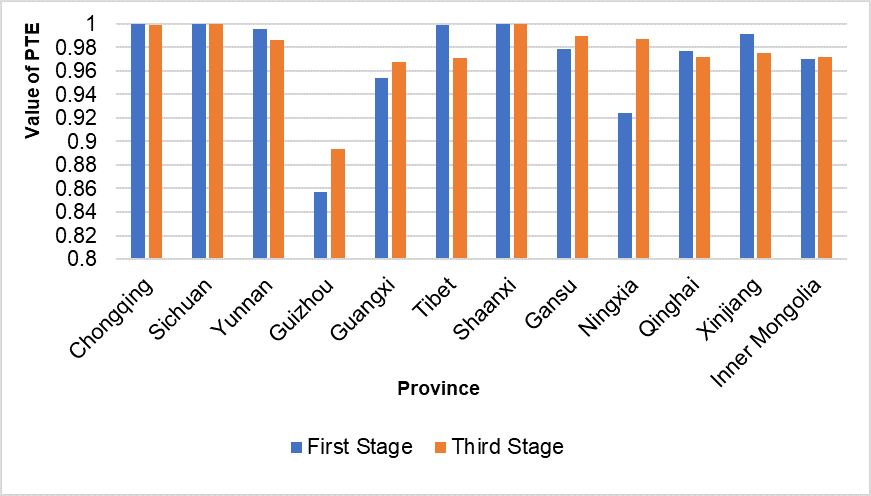

Supplement: S2 Fig — (TIF) [file pone.0325901.s002.tif]

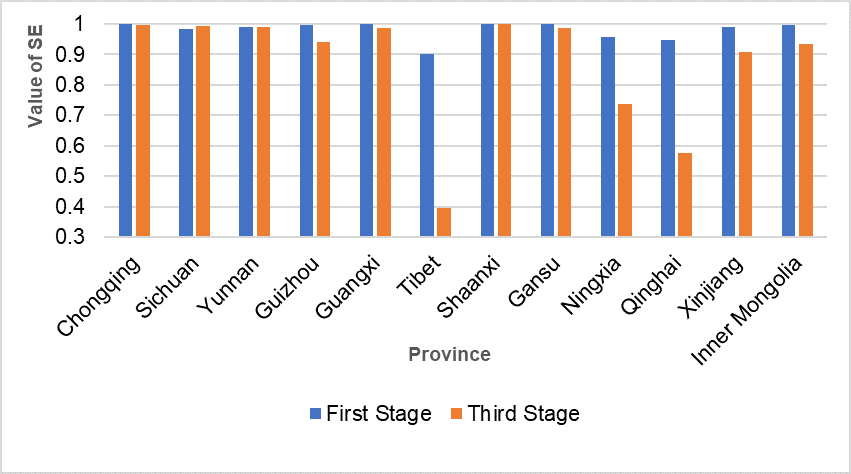

Supplement: S3 Fig — (TIF) [file pone.0325901.s003.tif]

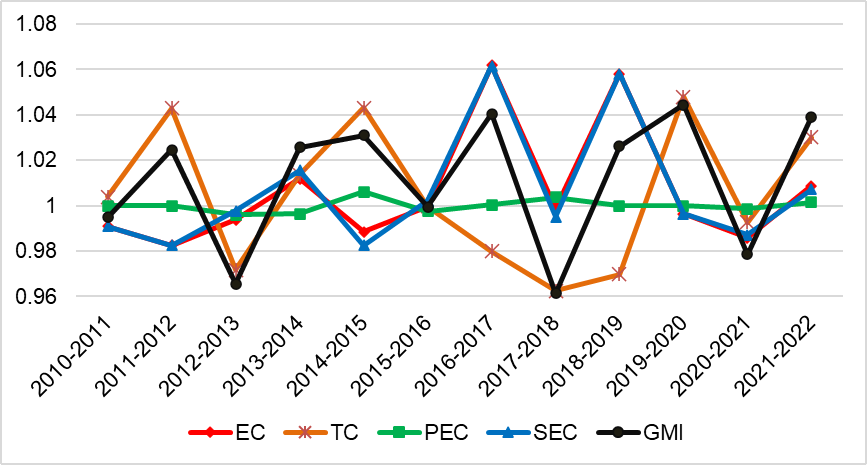

Supplement: S4 Fig — (TIF) [file pone.0325901.s004.tif]
