# Supplementary material for: Input-output efficiency, productivity dynamics, and determinants in western China’s higher education: A three-stage DEA, global Malmquist index, and Tobit model approach
Source: PLoS One. 2025 Jun 11;20(6):e0325901. doi: 10.1371/journal.pone.0325901 (PMC12157086; doi:10.1371/journal.pone.0325901)
Supplement: S1 Table — (DOCX) [file pone.0325901.s006.docx]

**S1 Table. Summary of Recent Relevant Literature on Input-output Indicators in Higher Education**

| **Scholars &Year of Publication** | **Input Variables** | **Output Variables** | **Study Area** |
| --- | --- | --- | --- |
| Sagarra et al. (2017) | Full-time equivalent faculty | Scopus papers | Mexico |
|  | Total enrolment | Graduates |  |
|  | First-joining graduates |  |  |
| Shamohammadi & Oh (2019) | Total number of full-time academic staff | Total annual publication count for each university × Annual average number of publication citations for each university/10 K | Korea |
|  | Total number of full-time nonacademic staff | Total annual international patent count for each university |  |
|  | Total amount of annual research funds | Total annual number of degrees awarded to undergraduate students in an academic year |  |
|  | Total number of undergraduate students enrolled | Total annual number of degrees awarded to students in an academic year |  |
|  | Total number of postgraduate students enrolled |  |  |
|  | Total amount of university’s fixed assets |  |  |
| Wang (2019) | Full-time equivalent faculty | Number of papers published in year | China |
|  | Number of faculty members of a university | Number of patent applications submitted |  |
|  | Number of postgraduates in a university | Revenue from knowledge transfer |  |
|  | Amount of research grant from the government |  |  |
|  | Amount of research grant from the industry |  |  |
| Liu et al. (2024) | Number of schools | Average students receiving higher education | China |
|  | Number of faculty members | Registered students in higher education schools |  |
|  | Number of R&D personnel | Publication on S&T |  |
|  | Funding received by higher education institutions | Science papers publications |  |
|  | Government expenditures for higher education |  |  |
|  | Fixed assets investment on higher education infrastructure |  |  |
| Song &Sun (2022) | Number of full-time faculty members | Full-time equivalent students | China |
|  | Number of administrative staff | Full-time equivalent graduates |  |
|  | Number of auxiliary teaching staff | Number of degree recipients |  |
|  | Number of service and maintenance staff | Average number of college students per 100,000 population |  |
|  | Full-time equivalent R&D personnel | Number of R&D projects |  |
|  | Total educational revenue | Number of published scientific papers |  |
|  | Expenditure on educational operations | Number of published scientific monographs |  |
|  | Infrastructure construction expenditure | National-level achievement awards |  |
|  | R&D expenditure | Number of valid invention patents |  |
|  | Total land area of the institution | Number of applied R&D projects |  |
|  | Total value of fixed assets | Number of science and technology service projects |  |
|  | Total building area | Actual revenue from technology transfer contracts |  |
|  | Total number of library holdings | Revenue from patent ownership transfers and licensing |  |
