# Supplementary material for: Input-output efficiency, productivity dynamics, and determinants in western China’s higher education: A three-stage DEA, global Malmquist index, and Tobit model approach
Source: PLoS One. 2025 Jun 11;20(6):e0325901. doi: 10.1371/journal.pone.0325901 (PMC12157086; doi:10.1371/journal.pone.0325901)
Supplement: S2 Table — (DOCX) [file pone.0325901.s007.docx]

**S2 Table. Initial Pure Technical Efficiency of Higher Education in Western China (2010-2022)**

| **Province** | **2010** | **2011** | **2012** | **2013** | **2014** | **2015** | **2016** | **2017** | **2018** | **2019** | **2020** | **2021** | **2022** | **Mean** | **Rank** |
| --- | --- | --- | --- | --- | --- | --- | --- | --- | --- | --- | --- | --- | --- | --- | --- |
| **Chongqing** | 1.0000 | 1.0000 | 1.0000 | 1.0000 | 1.0000 | 1.0000 | 1.0000 | 1.0000 | 1.0000 | 1.0000 | 1.0000 | 1.0000 | 1.0000 | 1.0000 | 1 |
| **Sichuan** | 1.0000 | 1.0000 | 1.0000 | 1.0000 | 1.0000 | 1.0000 | 1.0000 | 1.0000 | 1.0000 | 1.0000 | 1.0000 | 1.0000 | 1.0000 | 1.0000 | 1 |
| **Yunnan** | 1.0000 | 1.0000 | 1.0000 | 1.0000 | 0.9878 | 1.0000 | 1.0000 | 1.0000 | 1.0000 | 0.9544 | 1.0000 | 1.0000 | 1.0000 | 0.9955 | 5 |
| **Guizhou** | 1.0000 | 0.9162 | 0.8965 | 0.7933 | 0.7939 | 0.8685 | 0.8659 | 0.9581 | 0.8670 | 0.7317 | 0.7998 | 0.8052 | 0.8424 | 0.8568 | 12 |
| **Guangxi** | 1.0000 | 1.0000 | 1.0000 | 1.0000 | 1.0000 | 0.9887 | 0.9678 | 0.9047 | 0.8251 | 0.8549 | 1.0000 | 0.8560 | 1.0000 | 0.9536 | 10 |
| **Tibet** | 1.0000 | 1.0000 | 1.0000 | 0.9877 | 1.0000 | 1.0000 | 1.0000 | 1.0000 | 1.0000 | 1.0000 | 1.0000 | 1.0000 | 1.0000 | 0.9991 | 4 |
| **Shaanxi** | 1.0000 | 1.0000 | 1.0000 | 1.0000 | 1.0000 | 1.0000 | 1.0000 | 1.0000 | 1.0000 | 1.0000 | 1.0000 | 1.0000 | 1.0000 | 1.0000 | 1 |
| **Gansu** | 1.0000 | 1.0000 | 1.0000 | 1.0000 | 1.0000 | 1.0000 | 1.0000 | 1.0000 | 0.9155 | 0.8722 | 0.9622 | 0.9708 | 1.0000 | 0.9785 | 7 |
| **Ningxia** | 1.0000 | 1.0000 | 1.0000 | 1.0000 | 0.9204 | 0.8703 | 0.8934 | 1.0000 | 0.8710 | 0.8913 | 0.8428 | 0.8085 | 0.9117 | 0.9238 | 11 |
| **Qinghai** | 1.0000 | 1.0000 | 1.0000 | 1.0000 | 0.9610 | 0.9750 | 0.9485 | 0.8674 | 0.9513 | 1.0000 | 1.0000 | 0.9957 | 1.0000 | 0.9768 | 8 |
| **Xinjiang** | 1.0000 | 1.0000 | 0.9855 | 0.9879 | 1.0000 | 1.0000 | 0.9660 | 0.9902 | 0.9530 | 1.0000 | 1.0000 | 1.0000 | 1.0000 | 0.9910 | 6 |
| **Inner Mongolia** | 1.0000 | 0.9799 | 0.9760 | 0.9849 | 1.0000 | 1.0000 | 1.0000 | 1.0000 | 0.9121 | 0.8932 | 0.9423 | 0.9202 | 1.0000 | 0.9699 | 9 |
