# Supplementary material for: Input-output efficiency, productivity dynamics, and determinants in western China’s higher education: A three-stage DEA, global Malmquist index, and Tobit model approach
Source: PLoS One. 2025 Jun 11;20(6):e0325901. doi: 10.1371/journal.pone.0325901 (PMC12157086; doi:10.1371/journal.pone.0325901)
Supplement: S3 Table — (DOCX) [file pone.0325901.s008.docx]

**S3 Table. Initial Scale Efficiency of Higher Education in Western China (2010-2022)**

| **Province** | **2010** | **2011** | **2012** | **2013** | **2014** | **2015** | **2016** | **2017** | **2018** | **2019** | **2020** | **2021** | **2022** | **Mean** | **Rank** |
| --- | --- | --- | --- | --- | --- | --- | --- | --- | --- | --- | --- | --- | --- | --- | --- |
| **Chongqing** | 1.0000 | 1.0000 | 1.0000 | 1.0000 | 1.0000 | 1.0000 | 1.0000 | 1.0000 | 1.0000 | 1.0000 | 1.0000 | 1.0000 | 1.0000 | 1.0000 | 1 |
| **Sichuan** | 1.0000 | 1.0000 | 0.9878 | 0.9633 | 1.0000 | 1.0000 | 0.9626 | 0.9792 | 0.9952 | 0.9725 | 0.9688 | 0.9527 | 1.0000 | 0.9832 | 9 |
| **Yunnan** | 1.0000 | 1.0000 | 1.0000 | 0.9423 | 0.9575 | 1.0000 | 1.0000 | 1.0000 | 1.0000 | 0.9879 | 0.9962 | 1.0000 | 1.0000 | 0.9911 | 7 |
| **Guizhou** | 1.0000 | 0.9964 | 0.9993 | 0.9977 | 0.9995 | 0.9993 | 0.9987 | 0.9982 | 0.9984 | 0.9966 | 0.9962 | 0.9957 | 0.9954 | 0.9978 | 5 |
| **Guangxi** | 1.0000 | 1.0000 | 1.0000 | 1.0000 | 1.0000 | 0.9967 | 0.9984 | 0.9988 | 0.9998 | 0.9988 | 0.9987 | 0.9969 | 1.0000 | 0.9991 | 3 |
| **Tibet** | 1.0000 | 0.9236 | 1.0000 | 0.9357 | 0.9178 | 0.8144 | 1.0000 | 0.9073 | 0.8493 | 0.8596 | 0.8745 | 0.8173 | 0.8188 | 0.9014 | 12 |
| **Shaanxi** | 1.0000 | 1.0000 | 1.0000 | 1.0000 | 1.0000 | 1.0000 | 1.0000 | 1.0000 | 1.0000 | 1.0000 | 1.0000 | 1.0000 | 1.0000 | 1.0000 | 1 |
| **Gansu** | 1.0000 | 1.0000 | 1.0000 | 1.0000 | 1.0000 | 1.0000 | 1.0000 | 1.0000 | 0.9954 | 0.9876 | 0.9976 | 0.9998 | 1.0000 | 0.9985 | 4 |
| **Ningxia** | 1.0000 | 1.0000 | 1.0000 | 1.0000 | 0.9747 | 0.9704 | 0.9569 | 0.9566 | 0.9378 | 0.9027 | 0.9048 | 0.9186 | 0.9172 | 0.9569 | 10 |
| **Qinghai** | 1.0000 | 1.0000 | 1.0000 | 1.0000 | 0.8790 | 0.8473 | 0.9493 | 0.8891 | 0.8833 | 0.9328 | 1.0000 | 0.9209 | 1.0000 | 0.9463 | 11 |
| **Xinjiang** | 1.0000 | 1.0000 | 0.9992 | 0.9983 | 0.9912 | 0.9982 | 0.9631 | 0.9567 | 0.9703 | 1.0000 | 1.0000 | 1.0000 | 1.0000 | 0.9905 | 8 |
| **Inner Mongolia** | 1.0000 | 0.9738 | 0.9797 | 0.9943 | 1.0000 | 1.0000 | 1.0000 | 0.9984 | 0.9979 | 0.9973 | 0.9998 | 0.9991 | 1.0000 | 0.9954 | 6 |
