# Supplementary material for: Input-output efficiency, productivity dynamics, and determinants in western China’s higher education: A three-stage DEA, global Malmquist index, and Tobit model approach
Source: PLoS One. 2025 Jun 11;20(6):e0325901. doi: 10.1371/journal.pone.0325901 (PMC12157086; doi:10.1371/journal.pone.0325901)
Supplement: S4 Table — (DOCX) [file pone.0325901.s009.docx]

**S4 Table. Adjusted Pure Technical Efficiency of Higher Education in Western China (2010-2022)**

| **Province** | **2010** | **2011** | **2012** | **2013** | **2014** | **2015** | **2016** | **2017** | **2018** | **2019** | **2020** | **2021** | **2022** | **Mean** | **Rank** |
| --- | --- | --- | --- | --- | --- | --- | --- | --- | --- | --- | --- | --- | --- | --- | --- |
| **Chongqing** | 1.0000 | 0.9892 | 1.0000 | 1.0000 | 1.0000 | 1.0000 | 1.0000 | 1.0000 | 1.0000 | 1.0000 | 1.0000 | 1.0000 | 1.0000 | 0.9992 | 3 |
| **Sichuan** | 1.0000 | 1.0000 | 1.0000 | 1.0000 | 1.0000 | 1.0000 | 1.0000 | 1.0000 | 1.0000 | 1.0000 | 1.0000 | 1.0000 | 1.0000 | 1.0000 | 1 |
| **Yunnan** | 1.0000 | 1.0000 | 1.0000 | 0.9327 | 0.9213 | 1.0000 | 1.0000 | 1.0000 | 1.0000 | 0.9702 | 1.0000 | 1.0000 | 1.0000 | 0.9865 | 6 |
| **Guizhou** | 1.0000 | 0.9667 | 0.9682 | 0.8978 | 0.8556 | 0.9427 | 0.9241 | 0.9002 | 0.8342 | 0.7566 | 0.8425 | 0.8631 | 0.8707 | 0.8940 | 12 |
| **Guangxi** | 1.0000 | 0.9990 | 1.0000 | 1.0000 | 1.0000 | 1.0000 | 0.9690 | 0.9342 | 0.8752 | 0.9123 | 1.0000 | 0.8910 | 1.0000 | 0.9677 | 11 |
| **Tibet** | 1.0000 | 0.9584 | 1.0000 | 0.9140 | 1.0000 | 1.0000 | 0.9422 | 1.0000 | 0.9125 | 0.9724 | 0.9387 | 1.0000 | 0.9838 | 0.9709 | 10 |
| **Shaanxi** | 1.0000 | 1.0000 | 1.0000 | 1.0000 | 1.0000 | 1.0000 | 1.0000 | 1.0000 | 1.0000 | 1.0000 | 1.0000 | 1.0000 | 1.0000 | 1.0000 | 1 |
| **Gansu** | 1.0000 | 0.9813 | 1.0000 | 1.0000 | 1.0000 | 1.0000 | 1.0000 | 1.0000 | 0.9603 | 0.9200 | 1.0000 | 1.0000 | 1.0000 | 0.9894 | 4 |
| **Ningxia** | 1.0000 | 1.0000 | 0.9785 | 1.0000 | 0.9223 | 0.9596 | 0.9992 | 1.0000 | 1.0000 | 1.0000 | 1.0000 | 0.9832 | 0.9873 | 0.9869 | 5 |
| **Qinghai** | 0.9560 | 0.9471 | 1.0000 | 0.9566 | 0.9145 | 0.9292 | 0.9348 | 1.0000 | 1.0000 | 1.0000 | 1.0000 | 1.0000 | 1.0000 | 0.9722 | 8 |
| **Xinjiang** | 0.9584 | 0.9093 | 0.9061 | 0.9226 | 1.0000 | 1.0000 | 0.9807 | 0.9990 | 1.0000 | 1.0000 | 1.0000 | 1.0000 | 1.0000 | 0.9751 | 7 |
| **Inner Mongolia** | 0.9778 | 0.8837 | 0.9109 | 0.9597 | 1.0000 | 1.0000 | 1.0000 | 1.0000 | 0.9640 | 0.9521 | 1.0000 | 0.9825 | 1.0000 | 0.9716 | 9 |
