# Supplementary material for: Input-output efficiency, productivity dynamics, and determinants in western China’s higher education: A three-stage DEA, global Malmquist index, and Tobit model approach
Source: PLoS One. 2025 Jun 11;20(6):e0325901. doi: 10.1371/journal.pone.0325901 (PMC12157086; doi:10.1371/journal.pone.0325901)
Supplement: S5 Table — (DOCX) [file pone.0325901.s010.docx]

**S5 Table. Adjusted Scale Efficiency of Higher Education in Western China (2010-2022)**

| **Province** | **2010** | **2011** | **2012** | **2013** | **2014** | **2015** | **2016** | **2017** | **2018** | **2019** | **2020** | **2021** | **2022** | **Mean** | **Rank** |
| --- | --- | --- | --- | --- | --- | --- | --- | --- | --- | --- | --- | --- | --- | --- | --- |
| **Chongqing** | 1.0000 | 0.9533 | 1.0000 | 0.9863 | 1.0000 | 1.0000 | 1.0000 | 1.0000 | 1.0000 | 1.0000 | 1.0000 | 1.0000 | 1.0000 | 0.9954 | 2 |
| **Sichuan** | 1.0000 | 1.0000 | 1.0000 | 0.9881 | 1.0000 | 1.0000 | 0.9843 | 1.0000 | 1.0000 | 0.9848 | 0.9895 | 0.9599 | 1.0000 | 0.9928 | 3 |
| **Yunnan** | 1.0000 | 0.9759 | 1.0000 | 0.8943 | 0.9870 | 1.0000 | 1.0000 | 1.0000 | 1.0000 | 0.9990 | 1.0000 | 1.0000 | 1.0000 | 0.9889 | 4 |
| **Guizhou** | 1.0000 | 0.9320 | 0.8891 | 0.8685 | 0.9159 | 0.9351 | 0.9428 | 0.9371 | 0.9598 | 0.9595 | 0.9640 | 0.9665 | 0.9718 | 0.9417 | 7 |
| **Guangxi** | 1.0000 | 0.9731 | 1.0000 | 0.9956 | 1.0000 | 0.9973 | 0.8949 | 0.9955 | 0.9939 | 0.9974 | 1.0000 | 0.9879 | 1.0000 | 0.9873 | 6 |
| **Tibet** | 0.3501 | 0.3290 | 0.3532 | 0.3526 | 0.3552 | 0.3728 | 0.3888 | 0.4322 | 0.4154 | 0.4443 | 0.4308 | 0.4408 | 0.4606 | 0.3943 | 12 |
| **Shaanxi** | 1.0000 | 1.0000 | 1.0000 | 1.0000 | 1.0000 | 1.0000 | 1.0000 | 1.0000 | 1.0000 | 1.0000 | 1.0000 | 1.0000 | 1.0000 | 1.0000 | 1 |
| **Gansu** | 0.9826 | 0.9642 | 1.0000 | 1.0000 | 1.0000 | 1.0000 | 1.0000 | 1.0000 | 0.9745 | 0.9459 | 0.9757 | 0.9929 | 1.0000 | 0.9874 | 5 |
| **Ningxia** | 0.7681 | 0.8688 | 0.7814 | 0.7198 | 0.7542 | 0.6287 | 0.6637 | 0.8289 | 0.6933 | 0.7264 | 0.7224 | 0.6969 | 0.7414 | 0.7380 | 10 |
| **Qinghai** | 0.3533 | 0.4328 | 0.4825 | 0.4688 | 0.4578 | 0.4719 | 0.5797 | 0.5561 | 0.5763 | 0.7112 | 0.8404 | 0.7432 | 0.8369 | 0.5778 | 11 |
| **Xinjiang** | 0.8215 | 0.8428 | 0.8363 | 0.8576 | 0.8599 | 0.9708 | 0.8714 | 0.8632 | 0.8953 | 1.0000 | 1.0000 | 1.0000 | 1.0000 | 0.9091 | 9 |
| **Inner Mongolia** | 0.7582 | 0.8433 | 0.8454 | 0.8629 | 0.9195 | 1.0000 | 1.0000 | 1.0000 | 0.9853 | 0.9810 | 1.0000 | 0.9611 | 1.0000 | 0.9351 | 8 |
